# CRISPR/Cas9‐mediated knockout of six glycosyltransferase genes in *Nicotiana benthamiana* for the production of recombinant proteins lacking β‐1,2‐xylose and core α‐1,3‐fucose

**DOI:** 10.1111/pbi.12981

**Published:** 2018-08-11

**Authors:** Julia Jansing, Markus Sack, Sruthy Maria Augustine, Rainer Fischer, Luisa Bortesi

**Affiliations:** ^1^ Department for Molecular Biotechnology RWTH Aachen University Aachen Germany; ^2^ Present address: Indiana Biosciences Research Institute Indianapolis IN USA; ^3^ Present address: Aachen‐Maastricht Institute for Biobased Materials Maastricht University Geleen The Netherlands

**Keywords:** CRISPR/Cas9, gene knockout, α‐1,3‐fucosyltransferase, β‐1,2‐xylosyltransferase, glyco‐engineering, molecular farming

## Abstract

Plants offer fast, flexible and easily scalable alternative platforms for the production of pharmaceutical proteins, but differences between plant and mammalian N‐linked glycans, including the presence of β‐1,2‐xylose and core α‐1,3‐fucose residues in plants, can affect the activity, potency and immunogenicity of plant‐derived proteins. *Nicotiana benthamiana* is widely used for the transient expression of recombinant proteins so it is desirable to modify the endogenous N‐glycosylation machinery to allow the synthesis of complex N‐glycans lacking β‐1,2‐xylose and core α‐1,3‐fucose. Here, we used multiplex CRISPR/Cas9 genome editing to generate *N. benthamiana* production lines deficient in plant‐specific α‐1,3‐fucosyltransferase and β‐1,2‐xylosyltransferase activity, reflecting the mutation of six different genes. We confirmed the functional gene knockouts by Sanger sequencing and mass spectrometry‐based N‐glycan analysis of endogenous proteins and the recombinant monoclonal antibody 2G12. Furthermore, we compared the CD64‐binding affinity of 2G12 glycovariants produced in wild‐type *N. benthamiana*, the newly generated FX‐KO line, and Chinese hamster ovary (CHO) cells, confirming that the glyco‐engineered antibody performed as well as its CHO‐produced counterpart.

## Introduction

Plants have been used for the production of recombinant proteins for almost 30 years (Hiatt *et al*., 1989) and several technical, veterinary and pharmaceutical plant‐made proteins are now commercially available (Sack *et al*., 2015). In addition to versatility and speed, one of the main strengths of plant‐based production platforms is their ability to carry out complex post‐translational modifications, particularly the addition of complex N‐glycans related to the structures found on native mammalian proteins (Schähs *et al*., 2007). However, there are various differences between plant and mammalian N‐glycans, e.g. plant N‐glycans lack sialic acid and core α‐1,6‐fucose residues, but β‐1,2‐xylose and core α‐1,3‐fucose are present (Lerouge *et al*., 1998). The potential immunogenicity of the plant‐specific N‐glycans has been discussed extensively in the context of molecular farming (Bardor *et al*., 2003; Gomord *et al*., 2010; Shaaltiel and Tekoah, 2016), and although there are no conclusive data questioning the safety of plant‐made pharmaceuticals (Gomord *et al*., 2010; Rup *et al*., 2017; Santos *et al*., 2016; Tekoah *et al*., 2015; Ward *et al*., 2014), their elimination is desirable to produce glycoproteins lacking non‐human epitopes.

N‐glycosylation affects many properties of recombinant glycoproteins produced *in planta*. For example, plant‐made antibodies for passive immunization are as efficient as their mammalian counterparts in virus neutralization assays, but have a shorter half‐life in the blood due to a higher clearance rate (Ko *et al*., 2003). Removal of the core fucose residue—be it the mammalian α‐1,6‐fucose or the plant α‐1,3‐fucose—from the N‐glycan of an antibody led to a 20–50‐fold increase in antibody‐dependent cellular cytotoxicity (Cox *et al*., 2006; Loos *et al*., 2015; Shields *et al*., 2002; Zeitlin *et al*., 2011). Furthermore, the first plant‐made pharmaceutical protein to be approved by the FDA for human parenteral administration (taliglucerase alfa, proprietary name Elelyso, produced by Protalix Biotherapeutics as a replacement therapy of Gaucher's disease) benefits from terminal mannose residues on the α‐1,3‐fucose‐ and β‐1,2‐xylose‐containing N‐glycan structures generated in plant cell vacuoles (Tekoah *et al*., 2013). Exposed mannose residues are required for the efficient uptake of the enzyme into macrophages but terminal sialic acid residues are added when the equivalent product is made in mammalian cells, and these residues must be trimmed off *in vitro* before formulation (Grabowski *et al*., 2014; Mor, 2015). These examples demonstrate the ambiguity of N‐glycosylation in plant‐made pharmaceutical proteins: in some cases, the plant N‐glycans are beneficial and in other cases they are detrimental.

Fortunately, we can modify the genomes of plants used for recombinant protein production in order to tweak their N‐glycosylation machinery (Loos and Steinkellner, 2014). The first report describing the elimination of β‐1,2‐xylose and α‐1,3‐fucose from N‐glycans in the model plant *Arabidopsis thaliana* made use of the random T‐DNA insertion mutant collection (Alonso *et al*., 2003) to identify lines mutated in the single β‐1,2‐xylosyltransferase (*XylT*) and two α‐1,3‐fucosyltransferase (*FucT*) genes, verify their inactivation, and then cross them to generate triple knockout *A. thaliana* lines. The resulting lines were viable and had a normal phenotype despite their inability to transfer β‐1,2‐xylose and α‐1,3‐fucose to their N‐glycans (Strasser *et al*., 2004). Such comprehensive mutant collections do not exist for any other plant species used for recombinant protein production, so more direct methods are required. In the moss *Physcomitrella patens*, targeted integration of the human β‐1,4‐galactosyltransferase gene was used to knockout the *FucT* and *XylT* genes in order to humanize the N‐glycosylation machinery (Huether *et al*., 2005). This approach relies on homologous recombination, which is highly efficient in moss, but much less so in higher plants. Consequently, in the common duckweed (*Lemna minor*) (Cox *et al*., 2006), alfalfa (*Medicago sativa*) (Sourrouille *et al*., 2008) and the tobacco‐like species *Nicotiana benthamiana* (Strasser *et al*., 2008), an RNAi‐mediated knockdown approach was used to reduce plant‐specific glycosyltransferase activities. The *N. benthamiana* ΔXT/FT RNAi line developed almost 10 years ago (Strasser *et al*., 2008) has been a popular production variety for pharmaceutical proteins (Castilho *et al*., 2011; Dent *et al*., 2016; Olinger *et al*., 2012), even though this and other RNAi lines still have the capacity to synthesize α‐1,3‐fucose and β‐1,2‐xylose, albeit to a lesser extent than their wild‐type counterparts. Random mutagenesis by exposure to ethylmethanesulfonate (EMS) was used to develop *N. benthamiana* lines with point mutations in two *XylT* and five *FucT* genes, but 19%–34% of N‐glycans on endogenous leaf proteins in the 5KO (*FucT*) and 7KO (*FucT* + *XylT*) lines still contained α‐1,3‐fucose. By super‐transforming the 7KO lines with a *FucT* RNAi construct, the relative level of N‐glycans containing core α‐1,3‐fucose was reduced to 0.6%–0.9%, but α‐1,3‐fucosyltransferase activity was not fully eliminated (Weterings and Van Eldik, 2013).

With the advent of designer nucleases such as zinc finger nucleases (ZFNs) (Kim *et al*., 1996), transcription activator‐like effector nucleases (TALENs) (Christian *et al*., 2010), and most recently CRISPR‐related nucleases like Cas9 (Jinek *et al*., 2012) and Cpf1 (Zetsche *et al*., 2015), it has become increasingly straightforward to knock out the functions of multiple genes. In *N. benthamiana*, the two *XylT* genes and two of the five *FucT* genes were knocked out with TALENs to completely eliminate the β‐1,2‐xylosyltransferase activity and reduce core α‐1,3‐fucosyltransferase activity by 60%, the latter confirming that the other *FucT* genes would need to be targeted to eliminate FucT activity completely (Li *et al*., 2016). Two recent publications describe the successful multiplex CRISPR‐mediated knockout of four or five *FucT* genes and two *XylT* genes in *Nicotiana tabacum* BY‐2 suspension cells (Hanania *et al*., 2017; Mercx *et al*., 2017), resulting in the absence of α‐1,3‐fucose and β‐1,2‐xylose in endogenous and recombinant proteins, and no differences in cell morphology or growth between the knockout and wild‐type lines. However, to the best of our knowledge, the comprehensive knockout of all active *XylT* and *FucT* genes in intact *N. benthamiana* plants has not been achieved thus far. Here, we report the highly efficient multiplex knockout of two *XylT* and four *FucT* genes in *N. benthamiana* using CRISPR/Cas9 to generate lines for the production of recombinant proteins completely devoid of core α‐1,3‐fucose and/or β‐1,2‐xylose residues.

## Results

### Identification of α‐1,3‐fucosyltransferase and β‐1,2‐xylosyltransferase target genes

**Figure 1:**
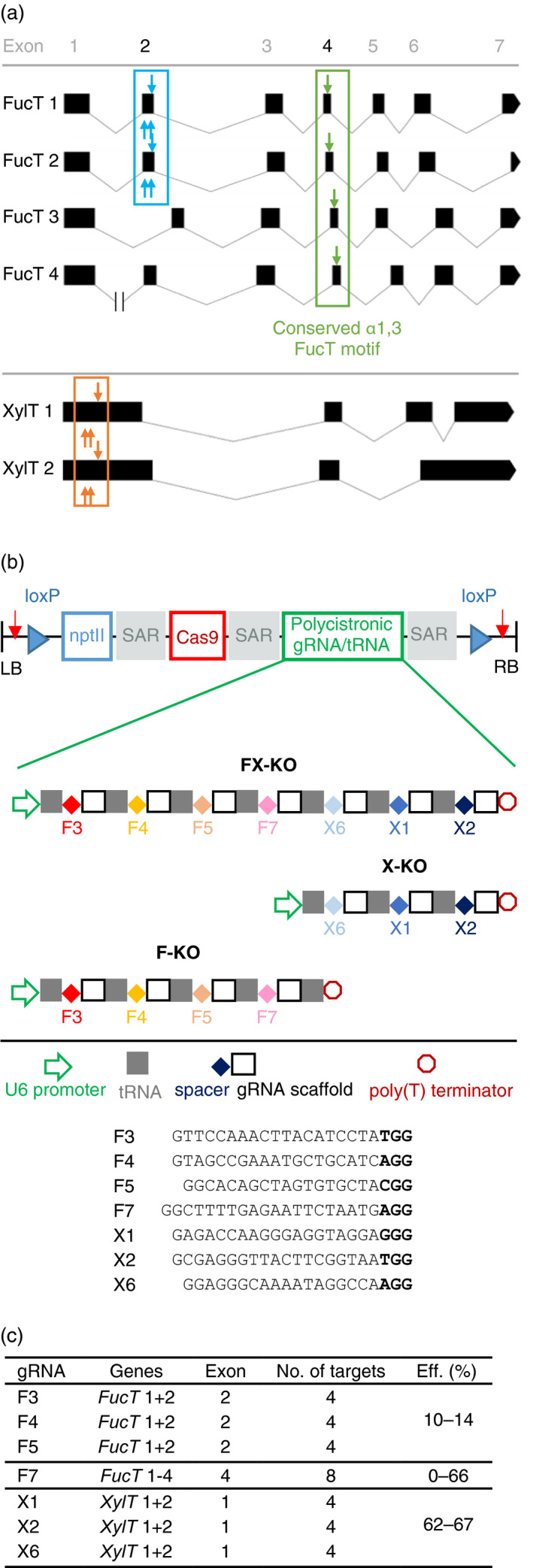
*FucT* 1‐4 and XylT 1 + 2 gene structure, schematic of transformation constructs and gRNA properties. (a) Structure of the four *FucT* genes targeted by four gRNAs and the two *XylT* genes targeted by three gRNAs (http://wormweb.org/exonintron). The *FucT* genes have seven exons, and conserved intron borders. *FucT* 2 has a premature stop codon in exon 7 resulting in a loss of 41 amino acids from the translated protein. *FucT* 4 has an unusually long intron 1 of ~7.8 kb. Three gRNAs (indicated by blue arrows) targeted exon 2 of *FucT* 1 and 2 (blue box), and one gRNA (green arrow) targeted the conserved catalytic motif in exon 4 of *FucT* 1, 2, 3 and 4 (green box). The three *XylT*‐specific gRNAs target exon 1 of *XylT* 1 and 2 (orange box). Altogether, the coloured boxes indicate the eight exons targeted by the selected gRNAs, resulting in a total of 16 targeted exons when both alleles of the six genes are considered. (b) The knockout constructs were flanked by left and right T‐DNA borders (LB and RB). To facilitate the removal of the construct from the plant genome, *loxP* sites (blue triangles) and human gRNA target sequences (red arrows) were included. For the selection of transformed plants on kanamycin, we included a neomycin phosphotransferase gene (*nptII*). The plant‐codon‐optimized *cas9* gene was controlled by a hybrid 35SPPDK promoter, and the corresponding polycistronic tRNA/gRNA gene (PTG) was controlled by the *A. thaliana* U6 promoter. The three genes were separated by scaffold attachment regions (SARs). In the three constructs F‐KO, X‐KO and FX‐KO, four, three and seven gRNAs are expressed from the PTG, respectively, and the gRNA sequences are provided including the PAM in bold. Not drawn to scale. (c) Properties of the gRNAs, their target genes and exons, the number of genomic targets, and their mutation efficiencies (Eff.) in T_0_ plants.

Two *N. benthamiana* β‐1,2‐xylosyltransferase (*XylT*) genes and five α‐1,3‐fucosyltransferase (*FucT*) genes with two alleles each were identified by literature research (Li *et al*., 2016; Strasser *et al*., 2008; Weterings and Van Eldik, 2013) and were used as BLAST queries against the draft *N. benthamiana* genome sequence (Bombarely *et al*., 2012) from the Sol Genomics Network (https://solgenomics.net; (Fernandez‐Pozo *et al*., 2015) and the Queensland University of Technology (http://benthgenome.qut.edu.au/; (Naim *et al*., 2012). Based on the sequences identified in the draft genome, specific primer pairs (Table S1) were designed for exon 1 (*XylT*) or exons 1, 2 and 4 (*FucT*) and were used to amplify and re‐sequence the regions of interest from *N. benthamiana* genomic DNA. The resulting sequences matched the published sequences with accession numbers Niben101Scf04551 (*XylT* 1), Niben101Scf04205 (*XylT* 2), Niben101Scf01272 (*FucT* 1), Niben101Scf02631 (*FucT* 2), Niben101Scf05494 (*FucT* 3), Niben101Scf17626 (*FucT* 4) and Niben101Scf05447 (*FucT* 5). The intron borders were found to be conserved in *FucT* 1, 2, 3 and 4 (Figure 1a), but not the intronless gene *FucT* 5, which also contains a Y288D amino acid substitution in the highly conserved fucosyltransferase motif. A mutation of that particular Tyrosine residue of a human FucT VI has been shown to completely inactivate the enzyme (Jost *et al*., 2005). *FucT* 5 also lacks a TATA box, indicating it is likely to be an inactive pseudogene (Weterings and Van Eldik, 2013). Accordingly, *FucT* 5 was not included in our knockout strategy.

### Design, evaluation and selection of gRNAs

**Figure 2:**
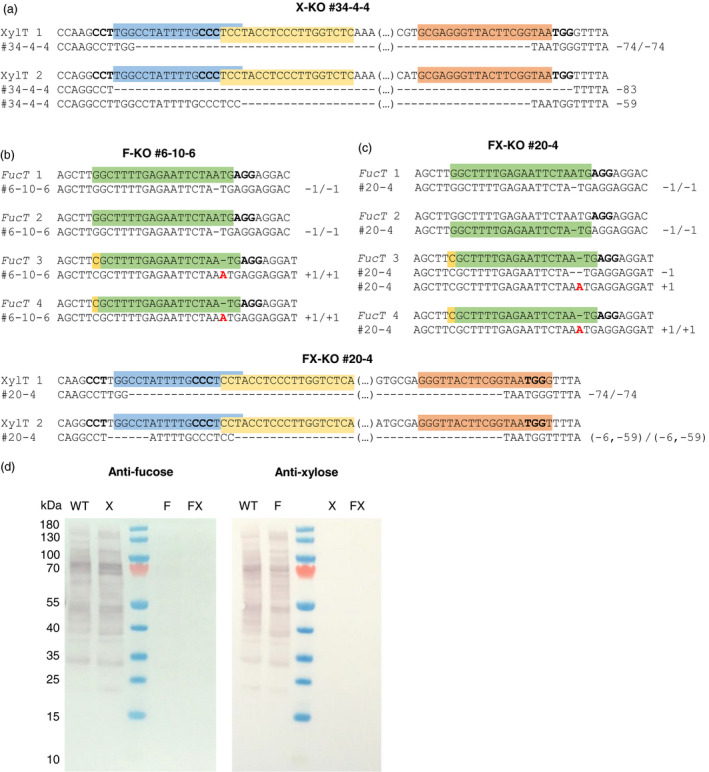
Sequencing and Western blot analysis for the T_2_ lines X‐KO #34‐4‐4 and F‐KO #6‐10‐6 and the F_2_ line FX‐KO #20‐4. (a) Mutations in *XylT* 1 and 2 of X‐KO #34‐4‐4 as identified by Sanger sequencing of PCR amplicons (*XylT* 1) and TOPO‐cloned PCR products (*XylT* 2). The corresponding wild‐type sequence is shown above, the gRNA target sequences are indicated by coloured boxes, and the PAM sequences are shown in bold. (b) Mutations in *FucT* 1, 2, 3 and 4 of F‐KO #6‐10‐6. The PAM‐distal mismatch between the gRNA and *Fuc*T 3 and 4 is highlighted in yellow. (c) Mutations in *FucT* 1‐4 and *XylT* 1 and 2 of FX‐KO #20‐4. Five of six genes carry homozygous mutations, with the exception of *FucT* 3 which has biallelic mutations. (d) Western blot of FX‐KO #20‐4 in comparison to *N. benthamiana* wild type, F‐KO #6‐10‐6 and X‐KO #34‐4‐4. ~10 µg of total soluble protein were loaded for each sample. Anti‐fucose blot: 1st antibody rabbit‐anti‐α1,3‐fucose (1 : 10 000), 2nd antibody goat‐anti‐rabbit H+L AP‐labelled (1 : 10 000, pre‐absorbed). Anti‐xylose blot: 1st antibody rabbit‐anti‐β1,2‐xylose (1 : 5000), 2nd antibody goat‐anti‐rabbit H+L AP‐labelled (1 : 10 000, pre‐absorbed).

The exon sequences were aligned to identify regions of homology that could be targeted by the same guide RNAs (gRNAs). Candidate gRNAs were evaluated for potential off‐targets, and six gRNAs each were designed to target the first and second exon of *XylT* 1 + 2 and *FucT* 1 + 2 but no other coding sequences in the genome. The gRNAs were transiently co‐expressed with Cas9 in *N. benthamiana* leaves, the genomic DNA was extracted after 5 days, the targeted regions amplified using gene‐specific primers (Table S2), and the mutation frequencies determined by next‐generation sequencing (Appendix S1, Table S3). We found that 0%–6% of the analysed amplicons were mutated at the anticipated target site, and the three most efficient gRNAs for both *XylT* 1 + 2 and *FucT* 1 + 2 were selected to be used in the knockout constructs for stable transformation (Figure 1b). At a later time, the publication by Li *et al*. (2016), showing that knocking out *FucT* 1 + 2 by TALENs does not completely abolish FucT activity, brought the genes *FucT* 3, 4 and 5 to our attention. *FucT* 3, 4 and 5 cluster together and have low nucleotide sequence identity with *FucT* 1 and 2, but the genes share the conserved catalytic fucosyltransferase motif in exon 4 (Jost *et al*., 2005). A single gRNA (F7) targeting this motif in the *FucT* 1–4 genes was therefore identified (a “silver bullet”), and included in the knockout constructs without testing its activity (Figure 1a, b). This gRNA was a perfect match for the target in *FucT* 1 and 2 but had a PAM‐distal G → C mismatch in *FucT* 3 and 4 (Figure 2b) which should be tolerated by Cas9 but might reduce the Cas9 nuclease activity in these two genes.

### Design and cloning of the multiplex knockout constructs for plant transformation

Three constructs for the generation of stable transgenic plants were prepared (Figure 1b): F‐KO (targeting four *FucT* genes), X‐KO (targeting two *XylT* genes) and FX‐KO (targeting four *FucT* genes and two *XylT* genes). Using synthetic polycistronic tRNA‐gRNA (PTG) cassettes (Xie *et al*., 2015), 3–7 gRNAs were included in each of the three constructs to facilitate the multiplex knockout of up to six genes. The *cas9* cassette was located upstream of the gRNA cassette and was separated from it by a scaffold attachment region (SAR). Next to the left and right borders (LB and RB), highly active human gRNA target sequences with no potential off‐target sites in the *N. benthamiana* genome, as well as *loxP* sites, were included to make the constructs excisable with either Cas9 or Cre recombinase in case segregating the transgene(s) from the final lines was not possible. After construct validation by restriction digest and Sanger sequencing, the knockout constructs were used to transform *Agrobacterium tumefaciens*.

### Generation, screening and selection of genome‐edited plants

#### T_0_ generation

In two rounds of transformation, 57 X‐KO plants, 43 F‐KO plants and 74 FX‐KO plants were regenerated and screened for mutations in the target genes by the Sanger sequencing of PCR amplicons. Gene‐specific primer pairs were used to separately amplify homologous target sequences, e.g. from *XylT* 1 and 2. A high percentage of plants (80%, 83% and 73%) showed evidence for Cas9‐mediated editing in at least one of the 4, 12 or 16 targeted exons respectively (Figure 1a, c). The mutations identified by sequencing mostly consisted of +1 insertions or short deletions, but some larger deletions of up to 371 bp were also found. Almost half of the mutated plants had mosaic mutation patterns in one or more of the target genes. The efficiencies of the *FucT*‐ and *XylT*‐specific gRNAs varied (Figure 1c): the three gRNAs targeting *XylT* 1 and 2 caused mutations in 62%–67% of the plants, whereas only 10%–14% of plants were mutated in exon 2 of *FucT* 1 and 2 targeted by three previously tested gRNAs. Unexpectedly, the ‘silver bullet’ gRNA F7 targeting the conserved fucosyltransferase motif in exon 4 of *FucT* 1–4 achieved different efficiencies in the F‐KO and FX‐KO plants. Whereas 83% of the F‐KO plants were mutated in at least one of eight targeted alleles, only 12% of the FX‐KO plants with at least one mutated allele were identified. Looking at the *FucT* 1, 2, 3 and 4 genes individually, we found mutations in 66%, 59%, 25% and 38% of 43 F‐KO plants, and in 5%, 8%, 3% and 0% of 74 FX‐KO plants respectively. Unfortunately, none of the plants transformed with the FX‐KO construct carrying gRNAs for both *XylT* and *FucT* had mutations in all six targeted genes. From the X‐KO construct, 10 plants with at least monoallelic mutations in both *XylT* genes were selected, as well as six plants with at least monoallelic mutations in the four targeted *FucT* genes from the F‐KO construct (Table S4). The selected lines were allowed to self‐pollinate and produce seeds.

#### T_1_ generation

Depending on the number of mutated genes and the zygosity of the mutations in the T_0_ line, 10–15 plants per line were grown for each of the 16 selected X‐KO and F‐KO genotypes. To quickly pre‐screen the resulting 185 plants for the inheritance and zygosity of the mutations detected in the T_0_ generation, high‐resolution melt analysis (HRMA) was carried out with gene‐specific primer pairs (Table S5) and lines with putative biallelic or homozygous mutations in all targeted genes were selected. This subgroup of plants was further tested by dot blot analysis with α‐1,3‐fucose‐specific and β‐1,2‐xylose‐specific antibodies (Figure S1a, b). Lines in which the dot blot signal intensity was lower than the wild‐type signal (or absent) were then genotyped by Sanger sequencing. We identified eight X‐KO lines with no evidence of XylT activity in the dot blots (Figure S1a, blue circles) and with complex biallelic or homozygous mutations in both *XylT* genes. Because the gene‐specific PCR amplicons covering two alleles were sequenced together, the resulting overlapping sequencing traces were difficult to analyse. The complexity and size of the mutations in both alleles made it difficult to specify the nature of the mutations in some cases, but we could determine that there was no wild‐type allele present without resorting to TOPO‐cloning. We also selected four F‐KO lines with mutations in six or seven of the eight *FucT* alleles (Table 1, Table S6, Figure S1b, blue circles), which displayed substantially reduced α‐1,3‐fucosyltransferase activity.

**Table 1:** CRISPR/Cas9‐induced mutations in the target genes of selected *N. benthamiana* knockout lines as determined by Sanger sequencing

Generation	Line	Gene	*FucT* 1	*FucT* 2	*FucT* 3	*FucT* 4	*XylT* 1	*XylT* 2
T_1_	F‐KO #6‐3	Mutation	+1/+1	+1	+1	+1/−6		
		Zygosity	Homo	Het	Het	Biall		
	F‐KO #6‐10	Mutation	−1/−6	−1	+1	+1/+1		
		Zygosity	Biall	Het	Het	Homo		
	F‐KO #35‐8	Mutation	−371/−371	−1	+1/+1	+1/+1		
		Zygosity	Homo	Het	Homo	Homo		
	F‐KO #35‐11	Mutation	−371/−371	−1	+1/+1	+1/+1		
		Zygosity	Homo	Het	Homo	Homo		
T_2_	**F‐KO #6‐10‐6**	Mutation	−1/−1	−1/−1	+1/+1	+1/+1		
		Zygosity	Homo	Homo	Homo	Homo		
	**X‐KO #34‐4‐4**	Mutation					−74/−74	−83/−59
		Zygosity					Homo	Biall
F_2_	**FX‐KO #20‐4**	Mutation	−1/−1	−1/−1	+1/−1	+1/+1	−74/−74	(−6, −59)/(−6, −59)
		Zygosity	Homo	Homo	Biall	Homo	Homo	Homo

The generation, type and name of line and the mutation status of the two, four or six target genes are provided. The length of the mutation is indicated, as well as the type (deletion −, insertion +). Biallelic and homozygous mutations are shown in the format mut/mut. Numbers in parentheses (mut,mut) indicate multiple mutations in one allele, i.e. mutations induced at different gRNA target sites. The full knockout lines confirmed by N‐glycan analysis are printed in bold.

#### T_2_ generation

Because the eight selected X‐KO lines were already mutated in all targeted genes in the T_1_ generation, only five plants per line were grown in the T_2_ generation. They were characterized by the Sanger sequencing of purified amplicons or TOPO‐cloned PCR products if necessary (Figure 2a), as well as dot blots (Figure S1c). Line X‐KO #34‐4‐4 was selected for further analysis of its N‐glycans (Figure S1c, blue circle).

The four selected F‐KO lines still had monoallelic mutations in one or two of the four targeted *FucT* genes in the T_1_ generation. Therefore, 50 plants for each line were grown in the T_2_ generation to ensure a good chance of identifying plants with homozygous mutations in all four target genes. The resulting 200 plants were pre‐screened by Sanger sequencing of *FucT* 2 exon 4, which had been heterozygous in all tested T_1_ plants. We identified 15–16 plants per line with homozygous mutations in *FucT* 2, and these were tested by dot blot using wild‐type and T_1_ samples as controls (Figure S1d). Surprisingly, there was no substantial further reduction in signal intensity compared to the T_1_ sample of each parental line, even though the T_1_ plant was not mutated in all the target alleles. Because the signal intensities were similar, six plants per line were selected randomly and genotyped by Sanger sequencing. Line F‐KO #6‐10‐6 with homozygous mutations in all four targeted *FucT* genes (Figure 2b, Figure S1d, blue circle) was selected for N‐glycan analysis.

### Analysis of total protein N‐glycans in selected F‐KO and X‐KO lines

Leaf material from the mature T_2_ plants F‐KO #6‐10‐6 and X‐KO #34‐4‐4 was treated with PNGase A to release the N‐glycans followed by MALDI‐TOF mass spectrometry analysis of the free glycan structures (Table 2, Figure S2a, b). N‐glycans were identified by their characteristic masses. The F‐KO sample comprised ~20% high‐mannose glycans and ~80% complex glycans, but no core‐fucosylated N‐glycans were detected (Figure S2a). A trace amount of Gn(FA)X was detected (the Lewis^a^ epitope with α‐1,4‐fucose), which would not be affected by knocking out α‐1,3‐fucosyltransferase. A similar picture emerged for the X‐KO sample, which comprised ~28% high‐mannose glycans, ~70% complex type glycans without xylose, and trace amounts of Gn(FA) and Gn(FA)F with the aforementioned Lewis^a^ epitope (Figure S2b). These results confirmed the complete knockout of all active *FucT* genes in the F‐KO line, and all active *XylT* genes in the X‐KO line.

**Table 2:** MALDI‐TOF‐MS analysis of total endogenous leaf protein N‐glycans released from lines F‐KO #6‐10‐6, X‐KO #34‐4‐4 and FX‐KO #20‐4

Glycan acronym	F‐KO #6‐10‐6	X‐KO #34‐4‐4	FX‐KO #20‐4
	% of total		
MUX	6.8		
MMX	43.1		
MGnX	17.2		
GnGnX	14.5		
Gn(FA)X	<1		
MMF		37.5	
MGnF		10.1	
GnGnF		13.5	
Gn(FA)F		<1	
Gn(FA)		<1	3.7
(FA)(FA)			2.5
MM		5.2	14.9
MGn		2.7	19.3
GnGn		2.9	37.0
Man4		3.8	
Man5	4.1	4.8	2.2
Man6	3.1	4.0	3.5
Man7	3.7	4.9	5.2
Man8	4.5	6.2	7.1
Man9	3.1	4.3	4.7

Symbols: X = β‐1,2‐xylosylated glycopeptides, F = α‐1,3‐fucosylated glycopeptides. Percentages describe the relative peak area of the N‐glycan as a ratio to the total peak area of the nine (F‐KO), 12 (X‐KO) or 10 (FX‐KO) most common N‐glycans. Minor peaks representing the Lewis^a^‐type N‐glycan variants were present in all samples, but for F‐KO and X‐KO the peak area was too small for quantification and thus not included in the calculation. The fucose in Lewis^a^‐type N‐glycans (FA) has an α‐1,4 linkage and its incorporation is thus not affected by the knockout of the α‐1,3‐fucosyltransferase genes. Glycan acronyms are based on proglycan nomenclature (http://www.proglycan.com/sites/default/public/pdf/nomen_2007.pdf).

### Transient expression and glycan analysis of the antibody 2G12 in selected T_2_ plants

To confirm that the selected knockout lines were suitable for the production of recombinant proteins lacking plant‐specific glycans, the antibody 2G12 (Trkola *et al*., 1996) was transiently expressed in young, detached leaves of lines F‐KO #6‐10‐6 and X‐KO #34‐4‐4, and wild‐type *N. benthamiana* plants, followed by purification and analysis for the identification and quantification of the heavy chain glycopeptide (Table 3). As expected, the results confirmed that no core α‐1,3‐fucose or β‐1,2‐xylose were present in the different N‐glycan structures commonly found on plant proteins. Consequently, the predominant glycoform shifted from GnGnXF in wild‐type leaves (62%) to GnGnX (67% in the F‐KO lines) or GnGnF (50% in the X‐KO lines). The absence of α‐1,3‐fucose or β‐1,2‐xylose indicated the successful functional knockout of all active gene variants in each of the lines.

**Table 3:** LC‐ESI‐MS analysis of N‐glycans released from antibody 2G12 transiently expressed in wild‐type *N. benthamiana* and the F‐KO #6‐10‐6, X‐KO #34‐4‐4 and FX‐KO #20‐4 lines

Glycan acronym	Wild type	F‐KO #6‐10‐6	X‐KO #34‐4‐4	FX‐KO #20‐4
	% of total			
Not glyc	14.5	14.0	14.0	15.8
Man7	2.0	1.3	1.8	2.6
Man8	5.3	1.3	2.3	7.8
Man9	1.5			3.7
GnM		0.5	1.9	2.8
GnGn	1.9	4.7	20.8	**65.4**
MMXF	1.6			
GnMXF	3.4			
GnGnXF	**62.4**			
MMX		0.9		
GnUX		1.4		
GnMX	0.5	7.2		
GnGnX	5.0	**67.6**		
MMF			3.0	
GnMF	0.3		4.9	
GnGnF	1.8		**50.3**	

Symbols: X =* *β*‐1,*2‐xylosylated glycopeptides, F = α‐1,3‐fucosylated glycopeptides. The dominant glycan structure in all samples is GnGn/X/F/XF (bold). Glycan acronyms are based on proglycan nomenclature (http://www.proglycan.com/sites/default/public/pdf/nomen_2007.pdf).

### F_1_ generation of FX‐KO

The confirmed knockout lines F‐KO #6‐10‐6 and X‐KO #34‐4‐4 were crossed to generate a heterozygous FX‐KO line. Because *N. benthamiana* is a self‐pollinating plant with delicate flowers, manual crossing is a challenging procedure. To ensure that the progeny of the F‐KO #6‐10‐6 x X‐KO #34‐4‐4 cross were indeed hybrids and not self‐pollination products, 20 plants were tested by dot blot with the β‐1,2‐xylose‐specific and α‐1,3‐fucose‐specific antibodies. The plants were expected to have heterozygous mutations in the six *XylT* and *FucT* genes and thus show reactivity in both dot blots. To our surprise, we identified FX‐KO #20, a line that tested negative in both dot blots (Figure S3a). Sanger sequencing of PCR amplicons showed that one mutated allele per gene was inherited from the parental lines and that new mutations had been induced in the wild‐type allele of all six genes (Figure S3b). Antibody 2G12 was transiently expressed in the detached leaves of line FX‐KO #20, and the N‐glycans of the purified antibody heavy chain were analysed as above (Figure S3c). No β‐1,2‐xylosylated N‐glycans were found, but 11% of the heavy chain glycopeptides contained α‐1,3‐fucose. N‐glycan analysis of the leaf proteome showed that ~50% of N‐glycans contained α‐1,3‐fucose, and that no β‐1,2‐xylose was present within the limit of detection (Figure S3d, e). This confirmed the complete knockout of the two *XylT* genes already in the F_1_ generation, and a substantial reduction in the level of α‐1,3‐fucosylation in comparison to wild‐type *N. benthamiana*. The remaining α‐1,3‐fucosyltransferase activity indicated that some of the newly induced mutations must have been somatic rather than germinal. To investigate the heritability of the mutations, the plant was allowed to self‐pollinate and the next generation was analysed.

### F_2_ generation of candidate line FX‐KO #20

Thirty F_2_ plants were grown from seeds obtained from the self‐pollination of line FX‐KO #20 and tested by dot blot analysis with the β‐1,2‐xylose‐specific and α‐1,3‐fucose‐specific antibodies. None of the extracts were recognized by the xylose‐specific antibody, and 10 were also not recognized by the fucose‐specific antibody. Selected lines were sequenced, and a plant with putative homozygous or biallelic mutations in all six target genes (Figure 2c) was used for N‐glycan analysis, focusing on the transiently expressed antibody 2G12 (Table 3) and the leaf proteome (Table 2, Figure S2c). The total soluble protein of the selected FX‐KO and the confirmed F‐KO and X‐KO lines was tested in Western blots with fucose‐ and xylose‐specific antibodies (Figure 2d). No α‐1,3‐fucosylated or β‐1,2‐xylosylated N‐glycans were detected, apparently confirming the complete germinal knockout of all six target genes and the successful generation of the desired *N. benthamiana* FX‐KO line.

These results confirm that both *XylT* genes had biallelic germinal mutations already in the F_1_ generation, because otherwise some of the F_2_ plants would have inherited wild‐type alleles and thus would have been recognized by the xylose‐specific antibody. On the other hand, the identification of fucose‐positive lines confirms that at least one of the *FucT* genes was not fully mutated in the germline of the F_1_ parent plant, and explains the presence of α‐1,3‐fucosylated N‐glycans in that plant.

### Functional analysis of antibody glycovariants

**Figure 3:**
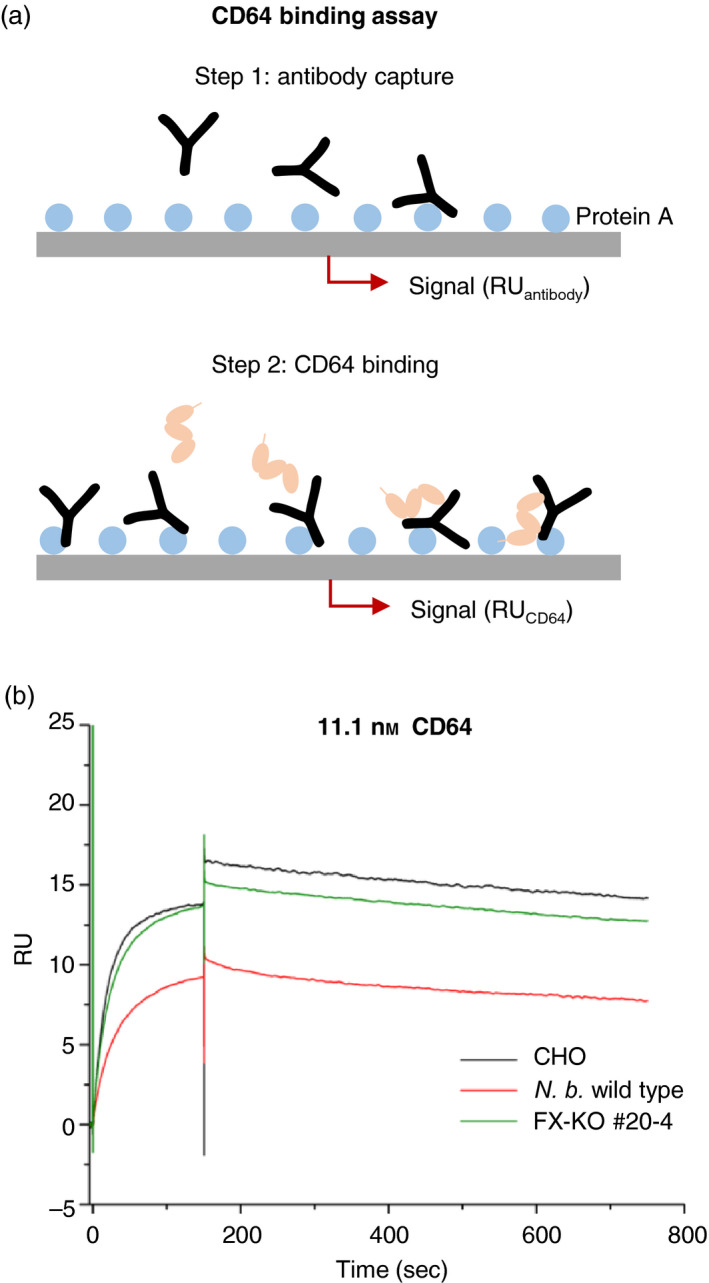
CD64 binding behaviour of 2G12 antibody glycovariants as determined by surface plasmon resonance spectroscopy. (a) Assay setup and procedure. Antibody preparations were bound to a protein A surface in previously determined dilutions to result in ~50 response units (RU) of bound antibody after 180 s contact time at a flow rate of 30 μL/min. The relative RU was determined after 10 min of washing with 1x HBS‐EP to precisely quantify the amount of antibody bound to the surface. CD64 was injected in one of eight dilutions (44.4–0.35 nm) and the binding kinetics determined by changes in the RU. Not drawn to scale. (b) CD64 binding curves of 2G12 glycovariants produced in wild‐type *N. benthamiana*, line FX‐KO #20‐4 and CHO cells. The curves show the RU changes upon injection of 11.1 nm CD64 dilution (*t* = 0 to 180 s) and the dissociation of CD64 from the antibody (*t* = 180 to 780 s). The RU values were corrected for the actual quantities of bound antibody and adjusted to run through the axis origin to improve comparability. The 2G12 expressed in wild‐type *N. benthamiana* showed a reduced affinity towards CD64 in comparison to CHO 2G12, but glyco‐engineered 2G12 expressed in line FX‐KO #20‐4 showed a similar affinity and comparable binding behaviour to the CHO version.

To assess the influence of the glycosylation profile on the properties of the recombinant antibody, we conducted a functional binding assay (Figure 3a) based on surface plasmon resonance (SPR) spectroscopy and compared the binding of different 2G12 glycoforms to CD64 (FCGR1A). We used 2G12 produced in Chines hamster ovary (CHO) cells, in wild‐type *N. benthamiana*, and in our FX‐KO line, captured equal amounts of purified antibody to a protein A surface, and measured the binding kinetics of eight CD64 dilutions (0.35–44.4 nm). As expected (Sack *et al*., 2007), the 2G12 produced in wild‐type plants showed a much lower affinity for CD64 than the corresponding antibody produced in CHO cells (Figure 3b). However, the affinity of the 2G12 produced in the FX‐KO line, lacking plant‐specific β‐1,2‐xylose and core α‐1,3‐fucose residues, was similar to that of its CHO counterpart (Figure 3b, Figure S5).

## Discussion

Ever since scientists became interested in using plants as a production platform for recombinant proteins, there was an awareness that the N‐glycans produced in plants could influence the behaviour of pharmaceutical glycoproteins *in vivo* (Parekh *et al*., 1989). Consequently, there has a been a lot of effort to modify the N‐glycosylation pathway in a variety of plant species, using T‐DNA insertion mutants (Strasser *et al*., 2004), RNAi (Cox *et al*., 2006; Sourrouille *et al*., 2008; Strasser *et al*., 2008), chemical mutagenesis (Weterings and Van Eldik, 2013) and, more recently, targeted nucleases (Hanania *et al*., 2017; Li *et al*., 2016; Mercx *et al*., 2017). *N. benthamiana* is of particular interest for molecular farming because the transient expression of proteins is fast and yields of more than 2 mg/g fresh leaf weight have been reported for antibodies (Zischewski *et al*., 2015). *N. benthamiana* is by far the most widely used species in different transient expression platforms, including the MagnICON system (Gleba *et al*., 2005), the pEAQ vector (Sainsbury *et al*., 2009) and the pTRA vector (Floss *et al*., 2008).

Here, we exploited the efficiency and multiplexing capability of the CRISPR/Cas9 system to knockout two β‐1,2‐xylosyltransferase and four α‐1,3‐fucosyltransferase genes in *N. benthamiana*. We established several F‐KO, X‐KO (by multiplexed gene knockout) and FX‐KO lines (by crossing F‐KO and X‐KO), verified the knockout of four (*XylT*), eight (*FucT*) or 12 (*XylT* and *FucT*) targeted alleles at the DNA level, and confirmed—within the detection limit of our assay methods—the absence of each glycosyltransferase activity by analysing both the N‐glycans of total endogenous proteins and the N‐glycans of the recombinant antibody 2G12 expressed transiently in the plants. To our knowledge, this is the first report of a complete knockout of FucT and XylT activity in *N. benthamiana*. We achieved high mutation frequencies in the T_0_ generation regenerated from leaf material infiltrated with the F‐KO, X‐KO and FX‐KO constructs: 73–83% of the T_0_ plants were mutated in at least one allele by at least one gRNA. Interestingly, the mutation efficiency of the gRNA targeting the conserved fucosyltransferase motif differed when expressed from the FX‐KO and F‐KO constructs, and varied from 0%–8% (FX‐KO) to 25%–66% (F‐KO). Although we anticipated a slightly lower efficiency for this gRNA in *FucT* 3 and *FucT* 4 due to the 5′ single‐nucleotide mismatch between the target and gRNA sequence (Figure 2b), it was puzzling to see such differences not between gene variants, but between constructs. This phenomenon is unlikely to reflect the dosages of the different gRNAs because the other gRNAs we used performed similarly in both constructs even though there were twice as many gRNAs expressed from the FX‐KO construct. One possible explanation for this difference is the position of the gRNA in the polycistronic gRNA‐tRNA gene (Figure 1b): in the F‐KO construct, this gRNA is in the very last position (4/4) and thus carries a poly(U) tail. In the FX‐KO construct, it is in the middle position (4/7) and does not have a poly(U) tail, but 1–4 nucleotides of the tRNA leader sequence are present instead (Xie *et al*., 2015). A similar effect was reported by (Xie *et al*., 2015) for their gRNA 2: when in position 2/2 in a PTG construct, the gRNA 2 induced biallelic mutations in 76% of plants, but when in position 2/4 only 24% of the plants had biallelic mutations. Taken together, these results suggest that gRNA expressed from the final position of a PTG construct carrying the poly(U) tail is considerably more efficient, but more comprehensive investigations are needed to confirm this.

The much lower mutation efficiency of the “silver bullet” gRNA as expressed from the FX‐KO construct—with not a single mutation in the *FucT* 4 gene among 74 T_0_ plants—made it impossible to identify a plant with at least monoallelic mutations in all six target genes, even though the overall mutation frequencies were high. One FX‐KO line with mutations in both *XylT* and two of the *FucT* genes was analysed in the next generation, but the mutations in the *FucT* genes were not inherited, presumably because they were somatic rather than germline mutations. The occurrence of mosaic or somatic mutations has been observed in the T_0_ generation of some genome‐edited plants (Jia *et al*., 2016; Nekrasov *et al*., 2013; Wang *et al*., 2015), which is why it is necessary to follow their inheritance through to the following generation. Indeed, we also observed the appearance of both somatic and germinal mutations in the hybrid F_1_ generation due to sustained Cas9 activity in the transgenic lines.

The *N. benthamiana* genome contains five α‐1,3‐fucosyltransferase genes, but our results show that the *FucT* 5 gene lacking a TATA box, intron and key catalytic motif does not need to be knocked out to prevent the synthesis of α‐1,3‐fucosylated N‐glycans. These data indicate that *FucT* 5 is likely to be an inactive pseudogene. Furthermore, we found that in plants with biallelic or homozygous mutations in *FucT* 1, 3 and 4, one intact allele of the *FucT* 2 gene is not sufficient to compensate for the FucT‐deficient phenotype. In dot blots probed with an α‐1,3‐fucose‐specific antibody, we did not observe any clear difference in signal intensity between the aforementioned plant lines with one intact *FucT* 2 allele and plants mutated in all eight alleles of the four *FucT* genes (Figure S1d, F‐KO #35‐8 vs. #35‐8‐13). This indicates that the *FucT* 2 gene—which is classed as a pseudogene in the NCBI GenBank database (accession number EF 562631)—contributes little or nothing to the overall α‐1,3‐fucosyltransferase activity in *N. benthamiana*, at least in leaves and under the experimental conditions we examined.

When analysing the endogenous N‐glycan composition of the F‐KO, X‐KO and FX‐KO lines, we observed similar ratios of complex or paucimannosidic (~75%–80%) to high‐mannose (~20%–25%) N‐glycans for the three lines (Table 2). The main glycoform in the FX‐KO line was GnGn in contrast to MMF in X‐KO and MMX in F‐KO. This difference can be explained with the findings of a recent study (Shin *et al*., 2017), which showed that the trimming of terminal GlcNAc residues in GnGn(X/F) glycofoms by a specific β‐hexosaminidase is promoted by the presence of either β‐1,2‐xylose or α‐1,3‐fucose. The absence of these sugar residues from the endogenous N‐glycans of the FX‐KO line thus results in less trimming and consequently a higher content of GnGn. For the 2G12 glycovariants, on the other hand, the main glycoforms were GnGn(F/X) variants in all tested samples (Table 3) as was the case for the 2G12 glycovariants expressed in the RNAi *N. benthamiana* lines by Strasser *et al*. (2008), likely because the antibody heavy chain is not a substrate for the β‐hexosaminidases present in the cell (Shin *et al*., 2017).

The F‐KO, X‐KO and FX‐KO lines we generated were devoid of β‐1,2‐xylose and/or α‐1,3‐fucose, the CRISPR‐induced mutations from T_1_ onwards were inherited by progeny plants, and the plants grew normally and expressed 2G12 at comparable levels following agroinfiltration (not shown). Together, the glyco‐engineered F‐KO, X‐KO and FX‐KO *N. benthamiana* lines offer considerable flexibility for the expression of glycoproteins in plants and will allow the effects of β‐1,2‐xylosylation and α‐1,3‐fucosylation on recombinant proteins to be studied in great detail, as shown for the 2G12 antibody. Furthermore, the lines can be used as a starting point for additional glyco‐engineering to reproduce human N‐glycosylation patterns, such as the introduction of pathways for the production, activation, transport and transfer of sialic acid to terminal galactose residues (Castilho *et al*., 2010).

From a plant physiology standpoint, it is also interesting to note that none of the three *N. benthamiana* knockout lines exhibited an obvious phenotype under greenhouse conditions, as also reported for *A. thaliana* triple mutants (Strasser *et al*., 2004). This suggests that the presence of the plant‐specific xylose and core‐fucose residues is not essential for survival, development or reproduction in *N. benthamiana*. Possibly, they are relevant in the context of biotic or abiotic stress, but further investigation is required to test this hypothesis.

We did not investigate off‐target effects because this was beyond the scope of our work, but the absence of an obvious phenotype indicates that no major unwanted genomic changes have occurred. To ensure genome stability in later generations, we designed the knockout constructs in such a way that allows their excision using CRISPR/Cas9 or the Cre/*loxP* system in case T‐DNA segregation is desired but not possible.

In conclusion, we have demonstrated that the highly efficient CRISPR/Cas9 system allows the rapid generation of *N. benthamiana* plants in which two, four or six endogenous genes are knocked out. We confirmed the inheritance of the induced mutations up to the T_3_ (F‐KO and X‐KO) and F_3_ (FX‐KO, see Figure S4) generations, as well as the intended xylose/core‐fucose‐free phenotypes by N‐glycan analysis. Finally, we showed that an antibody produced in the FX‐KO line displays higher affinity CD64 receptor binding than the same antibody produced in wild‐type plants, and comparable affinity to the gold standard produced in CHO cells. This highlights the importance of our knockout lines for the production of efficacious biopharmaceutical glycoproteins.

## Experimental procedures

### Resequencing the target genes and gRNA design

Genomic DNA was extracted from wild‐type *N. benthamiana Rdr1* insertion genotype (Bally *et al*., 2015; Spiegel *et al*., 2015) leaf material using the NucleoSpin Plant II kit (Macherey‐Nagel, Dueren, Germany). Potential target regions of the selected genes *XylT* 1, *XylT* 2, *FucT* 1, *FucT* 2, *FucT* 3, *FucT* 4 and *FucT* 5 were amplified from genomic DNA using the Expand High Fidelity PCR system (Roche, Basel, Switzerland), purified from agarose gels using the NucleoSpin Gel and PCR Clean‐up kit (Macherey‐Nagel) and sequenced using the Sanger method prior to gRNA design, with the primers listed in Table S1. All primers were purchased from Eurofins Genomics (Ebersberg, Germany). The sequences were aligned in three groups (*XylT* 1 and 2; *FucT* 1 and 2; *FucT* 3, 4 and 5) and the gRNAs were designed to target all genes in a group. CRISPR‐P v1.0 (Lei *et al*., 2014) was used to assess the potential off‐target sites of each gRNA. We set the allowed maximum off‐target score to 5 of 100 possible points, and excluded gRNAs whose 12‐nt seed region had a perfect match in a coding sequence elsewhere in the genome. In addition, all gRNA candidates were used as BLAST queries against the *N. benthamiana* genome scaffold v0.5 (http://benthgenome.qut.edu.au/) to identify further potential off‐targets.

### Cloning of CRISPR constructs for gRNA testing and stable transformation

The 12 gRNAs selected to be tested by transient expression were obtained as String DNA Fragments (Thermo Fisher Scientific/GeneArt, Regensburg, Germany), amplified by PCR using the primers U6‐AscI‐F (5′‐TTT GGC GCG CCA TAG CTG TTT GCC ATC GCT AC‐3′) and gRNA‐KpnI‐R (5′‐CGA GGT ACC TAA TGC CAA CTT TGT ACA AGA AAG C‐3′), digested with KpnI‐HF and PacI (New England Biolabs, Frankfurt am Main, Germany) and inserted into the binary pPAM vector (GenBank: AY027531.1) carrying a plant‐codon optimized *cas9* cassette with intron (Li *et al*., 2013) and a gRNA expression cassette under the control of the *A. thaliana* U6 promoter (Addgene #52254).

For the stable transformation of *N. benthamiana* plants, three variants of the knockout construct were prepared: one with three gRNAs targeting *XylT* 1 and 2 (X‐KO), one with four gRNAs targeting *FucT* 1–4 (F‐KO), and one with the seven gRNAs combined (FX‐KO). The three knockout constructs were based on the binary pPAM plasmid and carried an *npt*II cassette for positive selection on kanamycin, a plant‐codon optimized *cas9* cassette, and a synthetic polycistronic tRNA‐gRNA cassette (Xie *et al*., 2015), interspaced with SARs (Hall *et al*., 1991). For later excision of the construct from the plant genome, a human gRNA target (5′‐TTG GCA GGG GGT GGG AGG GA**A GG**‐3′, gRNA AS2 (Cho *et al*., 2014)) and *loxP* (Sternberg and Hamilton, 1981) sites were cloned next to the left and right borders of the T‐DNA. The three PTG cassettes and gRNA/*loxP* sequences were synthesized by GeneArt.

### Agroinfiltration

Electrocompetent *A. tumefaciens* GV3101::pMP90RK cells were transformed with purified and sequence‐verified plasmids for gRNA testing, stable transformation and 2G12 antibody expression. Selected clones were cultivated in YEB medium (0.5% (w/v) beef extract, 0.1% (w/v) yeast extract, 0.5% (w/v) peptone, 2 mm sterile MgSO_4_) with 50 mg/L kanamycin, 50 mg/L rifampicin and 100 mg/L carbenicillin at 26 °C and 160 rpm for 2 days. The OD_600_ of the culture was determined and diluted to a final value of 1 with fresh 2x infiltration medium (10% (w/v) sucrose, 0.4% (w/v) glucose, 0.1% (w/v) Ferty 2 MEGA, pH 5.6) and water. The resulting infiltration solution was induced with 200 μm acetosyringone for 30 min at room temperature, and injected into the undersides of young leaves of greenhouse‐grown 7‐week‐old intact plants (gRNA testing, stable transformation) or detached young leaves of 8–12‐week‐old plants (2G12 antibody expression). After incubation for 5–7 days in a phytochamber at 22 °C with a 16‐h photoperiod, the infiltrated material was harvested.

### Stable transformation and plant cultivation

Agroinfiltrated *N. benthamiana* leaves were harvested after 4–5 days, surface sterilized, rinsed three times with sterile water, cut into ~2‐cm² pieces and placed on shooting medium selection plates (MS II: MS medium, 1 mg/L 6‐benzylaminopurine, 0.1 mg/L naphthaleneacetic acid, 100 mg/L kanamycin, 200 mg/L cefotaxime, pH 5.8) and kept under controlled conditions (25 °C, 16‐h photoperiod). After 3–4 weeks, shoots appeared, were cut, transferred to MS III plates (MS medium, 100 mg/L kanamycin, 200 mg/L cefotaxime, pH 5.8) and incubated at 23–25 °C until roots formed after 10–14 days. The plantlets were then transferred into Weck jars containing MS III medium, incubated for 2 weeks and then transferred to soil.

### Extraction of genomic DNA

For next‐generation sequencing, high‐quality genomic DNA was extracted from infiltrated leaf material using the NucleoSpin Plant II kit. For direct sequencing and HRMA, genomic DNA was extracted using the alkaline lysis method (Xin *et al*., 2003). Briefly, a leaf disc with a diameter of ~3 mm was placed in 50 μL of buffer A (100 mm NaOH, 2% Tween‐20) and incubated at 95 °C for 10 min before adding 50 μL of buffer B (100 mm Tris‐HCl, 2 mm EDTA, pH 2.0) to adjust the pH. We then used 0.5–1 μL of the crude extract as a template for PCR or 0.25 μL as a template for HRMA in 20‐μL reaction mixes. The extracts were stored at 4 °C.

### Amplification of target regions and Sanger sequencing of amplicons

The gRNA target regions were amplified from 1 μL of genomic DNA extract using Q5 Hot Start High‐Fidelity 2x Master Mix (New England Biolabs) in a 20‐μL reaction, and the products purified either directly or from agarose gels using the NucleoSpin Gel and PCR Clean‐up kit, or by robot‐assisted PCR clean‐up with NucleoFast 96 PCR plates (Macherey‐Nagel). The amplicons were then sequenced in house using the Sanger method, and the results were analysed using Clone Manager v9 Professional (Scientific & Educational Software, Denver) and the online tool TIDE (Brinkman *et al*., 2014). When the sequencing results were ambiguous, the PCR products were cloned in pTOPO from the TOPO TA Cloning Kit (Thermo Fisher Scientific, Waltham) and 5–10 clones were sequenced individually for each sample. A detailed explanation of the analysis procedure can be found in Appendix S2 and Figure S6.

### High‐resolution melt analysis

For the initial screening of T_1_ generation plants, the targeted regions were amplified from 0.25 μL genomic DNA extract using iQ Sybr Green Supermix (Bio‐Rad, Munich, Germany) in a 20‐μL reaction volume (10 μL 2× Supermix, 0.5 μm forward primer, 0.5 μm reverse primer, 0.25 μL genomic DNA, 6 μL deionized water) in a LightCycler 480 Real‐Time PCR system (Roche Molecular Diagnostics, Pleasanton) (95 °C 3 min, 40× 95 °C 10 s, 60 °C 30 s, melting point determination 60–95 °C, 0.02 °C/cycle). The melting curves were analysed using the online tool uAnalyze v2.0 (Dwight *et al*., 2012). All amplicons were 100–150 bp in length, and were prepared using the primers in Table S5.

### Western Blot of total soluble protein from leaf material

Leaf samples were ground in two volumes of cold 1× PBS (137 mm NaCl, 2.7 mm KCl, 8.1 mm Na_2_HPO_4_, 1.5 mm KH_2_PO_4_, pH 7.4) and centrifuged for 10 min at 16 100 ***g*** and 4 °C. The protein content of the supernatant was quantifies using a NanoDrop ND1000 spectrophotometer (Thermo Fisher Scientific), and 10 μg TSP per lane used for reducing SDS‐PAGE and Western blots. The reducing SDS‐PAGE was performed as described in Appendix S4. After electrophoresis, the proteins were transferred to a nitrocellulose membrane (Thermo Fisher Scientific) in a blotting tank with blotting buffer (25 mm Tris, 192 mm glycine, 20% (v/v) methanol) for 1 h at 100 V. The membrane was then blocked for 1 h in 5% skim milk in TBS‐T (20 mm Tris, 150 mm NaCl, 0.05% (v/v) Tween‐20, pH 7.5), briefly washed, and then incubated with the primary antibody (Agrisera AB, Umea, Sweden) for 2 h in standard TBS‐T (anti‐β‐1,2‐xylose, 1 : 5000) or 1 h in high‐salt TBS‐T (anti‐α‐1,3‐fucose 1 : 10 000; high‐salt TBS‐T: 20 mm Tris, 500 mm NaCl, 0.1% Tween, pH 7.5). During incubation with the primary antibody, the secondary goat‐anti‐rabbit H+L AP‐labelled antibody (Jackson ImmunoResearch Laboratories, Westgrove) was pre‐absorbed to remove antibodies binding unspecifically to plant leaf extract. For this purpose, two gel lanes were loaded with a high concentration of wild‐type *N. benthamiana* leaf extract, blotted, blocked, and then incubated with a 1 : 10 000 dilution of the secondary antibody in standard TBS‐T. After 1.5–2 h of pre‐absorption, the antibody dilution was collected, the membrane briefly rinsed with high‐salt TBS‐T, and both fractions pooled to obtain a medium‐salt antibody solution.

After incubation with the primary antibody, the sample membranes were washed 3x in the standard (anti‐xylose) or high‐salt (anti‐fucose) TBS‐T, and then incubated with the pre‐absorbed secondary antibody for 1 h. The membranes were washed 3x in high‐salt TBS‐T and 1x in AP buffer, and then developed by adding 100 μL BCIP/NBT in 10 mL AP buffer and incubation in the dark at RT.

### Glycoprotein purification and analysis

The infiltrated frozen leaf material was weighed and extracted in 2–3 volumes of protein extraction buffer as described above using a mortar and pestle. The homogenate was filtered through Miracloth (Merck, Darmstadt, Germany), adjusted to pH 7.4 with 0.1 volumes of 1 m Tris (pH 8.0), and then centrifuged at 3220 ***g*** at 4 °C for 20 min. Protein A columns for affinity chromatography were prepared by pipetting 0.5 mL of Protein A Ceramic HyperD F (Pall, Dreieich, Germany) into a column and equilibrating with 10 column volumes of PBS. After loading the supernatant and washing with 10 column volumes of PBS, the bound antibodies were eluted in an elution buffer suitable for 2G12 (100 mm glycine, 100 mm fructose, pH 3.6). Seven eluate fractions of 500 μL were captured and buffered with 0.1 volumes of 1 m acetate buffer (pH 4.75). The protein content of the eluate was quantified using a NanoDrop ND1000 spectrophotometer (Thermo Fisher Scientific) and the fraction with the highest concentration was used for CD64 receptor binding assays. The samples were mixed with 4x NuPAGE LDS Sample Buffer, 10x NuPAGE Reducing Agent (Thermo Fisher Scientific) and incubated at 70 °C for 10 min. The samples were separated by SDS‐PAGE at 200V for 35 min using precast NuPAGE Novex 4%–12% Bis‐Tris protein gels and NuPAGE MES SDS Running Buffer (Thermo Fisher Scientific). After staining with Coomassie Brilliant Blue (Fairbanks *et al*., 1971; Wong *et al*., 2000), the antibody heavy chain bands from four lanes loaded with the same sample were pooled for N‐glycan analysis (Appendix S3) as previously described (Kolarich and Altmann, 2000; Strasser *et al*., 2008). Figure S5b shows the Coomassie‐stained gels of the 2G12 glycovariants.

### N‐glycan analysis of total endogenous leaf proteins

Several young leaves (0.5–1 g) from different parts of the plant were harvested and N‐glycans of endogenous proteins released by PNGase A were analysed by MALDI‐TOF mass spectrometry as previously described (Strasser *et al*., 2004, 2008).

### Comparison of antibody glycovariant binding kinetics to CD64 (FCGR1A) by surface plasmon resonance spectroscopy

Protein A‐purified antibody preparations (2G12 WT, 2G12 FX‐KO) and human monoclonal antibody 2G12 (Polymun Scientific, Klosterneuburg, Austria) were diluted in HPS‐EP buffer (10 mm HEPES, 150 mm NaCl, 3 mm EDTA, 0.05% Tween‐20, pH 7.4) for pre‐quantification on a protein A surface by surface plasmon resonance spectroscopy using Biacore T200 instrument (GE Healthcare, Freiburg, Germany). Based on the resulting response units (RU), the antibody samples were then diluted to yield 50 RU after 180 s contact with a 30 μL/s flow rate on the protein A surface. The lyophilized CD64 (FCGR1A) receptor (Sino Biological, Beijing, China) was reconstituted in water and serially diluted to 0.35, 0.7, 1.4, 2.8, 5.6, 11.1, 22.2 and 44.4 nm. To determine the binding kinetics between the antibody variants and CD64, ~50 RU of the antibody were bound to the protein A surface, and increasing concentrations of the receptor were injected with 180 s contact time, followed by 10 min dissociation. The resulting binding curves were analysed using the BIA evaluation software. The curves shown here were derived from the original spectrograms, corrected with the actual RU value (corrected RUkinetic = raw RUkinetic*(50 RUantibody/actual RUantibody), and normalized to start at the axis origin (*x* = 0/*y* = 0).

## Supporting information


**Figure S1** Dot blots for T_1_ and T_2_ X‐KO and F‐KO plants.
**Figure S2** Leaf proteome N‐glycan analysis for F‐KO #6‐10‐6, X‐KO #34‐4‐4 and FX‐KO #20‐4.
**Figure S3** Analysis of the hybrid F_1_ generation line FX‐KO #20.
**Figure S4** Dot blots of 50 F3 progeny plants of FX‐KO #20‐4.
**Figure S5** SPR assay for CD64 binding of 2G12 glycovariants.
**Figure S6** Examples for Sanger sequencing chromatograms.
**Table S1** Primers for the amplification and sequencing of genomic target regions.
**Table S2** Primers for the preparation of NGS samples.
**Table S3** Mutations frequencies of gRNAs *in transient*.
**Table S4** Mutations in selected F‐KO and X‐KO T_0_ lines.
**Table S5** Primers for HRMA analysis.
**Table S6** Mutations in selected F‐KO T_1_ plants.
**Appendix S1** Next‐generation sequencing.
**Appendix S2** Sanger sequencing chromatogram analysis.
**Appendix S3** Antibody N‐glycan analysis by LC‐ESI‐MS.
**Appendix S4** Dot blots of knockout candidate lines.
