# Supplementary material for: CRISPR/Cas9‐mediated knockout of six glycosyltransferase genes in Nicotiana benthamiana for the production of recombinant proteins lacking β‐1,2‐xylose and core α‐1,3‐fucose
Source: Plant Biotechnol J. 2018 Aug 11;17(2):350–61. doi: 10.1111/pbi.12981 (PMC6335070; doi:10.1111/pbi.12981)
Supplement: Supplementary file 1 — Figure S1 Dot blots for T1 and T2 X‐KO and F‐KO plants. Figure S2 Leaf proteome N‐glycan analysis for F‐KO #6‐10‐6, X‐KO #34‐4‐4 and FX‐KO #20‐4. Figure S3 Analysis of the hybrid F1 generation line FX‐KO #20. Figure S4 Dot blots of 50 F3 progeny plants of FX‐KO #20‐4. Figure S5 SPR assay for CD64 binding of 2G12 glycovariants. Figure S6 Examples for Sanger sequencing chromatograms. Table S1 Primers for the amplification and sequencing of genomic target regions. Table S2 Primers for the preparation of NGS samples. Table S3 Mutations frequencies of gRNAs in transient. Table S4 Mutations in selected F‐KO and X‐KO T0 lines. Table S5 Primers for HRMA analysis. Table S6 Mutations in selected F‐KO T1 plants. Appendix S1 Next‐generation sequencing. Appendix S2 Sanger sequencing chromatogram analysis. Appendix S3 Antibody N‐glycan analysis by LC‐ESI‐MS. Appendix S4 Dot blots of knockout candidate lines. [file PBI-17-350-s001.docx]

**Supplementary material**

**Suppl. Table 1**: Primers used for the amplification and sequencing of potential genomic target regions: *XylT* 1, 2 and *FucT* 1, 2, 3, 4 and 5.

| Name | Sequence (5ʹ→3ʹ) |
| --- | --- |
| XylT1 5ʹUTR | TACGGTAGGTGACGACCATC |
| XylT1 Int1 rev | GGCATCTAGTCGGGATATTC |
| XylT2 5ʹUTR | GAGAGGTTGTCCCGAAATAG |
| XylT2 Int1 rev | CTGCCAACATTCACAAAGTTC |
| FucT1 5ʹUTR | GTCTGTAGTTGACCTGTATTC |
| FucT1 Int1 rev | GAGAATTCATGCAGCCGACT |
| FucT1 Int1 fw | GGATAGAGGCGTAGTAGTAG |
| FucT1 Int2 rev | GACCTATAGCTGCCAATACG |
| FucT1 Int3 fw | GGTGTGGTGGACAAATCATC |
| FucT1 Int4 rev | AAGGCGCAAAGTCTTGGATG |
| FucT2 5ʹUTR | CCGTACCTGTGGGCATGAAC |
| FucT2 Int1 rev | GGAGTAGGCTGTATGAATCC |
| FucT2 Int1 fw | CCATGCTATGCGCTTGTTAC |
| FucT2 Int2 rev | CAGGGTCAGGGATGATTCAG |
| FucT2 Int3 fw | GACTCTTCCTTGGGTATCAC |
| FucT2 Int4 rev | TGTCCTTCCTGCAACTTC |
| FucT3 5ʹUTR | TCCAACTGTCCCACCAAATG |
| FucT3 Int1 rev | AGAGGTCACATGGCAACAAC |
| FucT3 Int1 fw | TCTCTTGCCTGTGTTCCTTC |
| FucT3 Int2 rev | CAGGTGCCAAATGTGGTGAG |
| FucT3 Int3 fw | AGAAGCTAGACCAGGGTTTG |
| FucT3 Int4 rev | GAGAAGGAGCAAAGTCTAGG |
| FucT4 5ʹUTR | CCCAACTGTCCCACCAAATG |
| FucT4 Int1 rev | GCTCCAAGGCTCCCTTTCTG |
| FucT4 Int1 fw | ACCAGTACCCGGTTATCATC |
| FucT4 Int2 rev | TTCTTGGAGTCGCTGGATTC |
| FucT4 Int3 fw | AGACCAGGTTTGTTGGAAAGG |
| FucT4 Int4 rev | GGTTGCATTTGGTCCAAAGG |
| FucT5 fw1 | TGCCTGACTTTCACTCCTAC |
| FucT5 rev1 | TGTAGCGCTTGAGAGTTTCC |
| FucT5 fw2 | TTGTGGTGCTCGTAACTTCC |
| FucT5 rev2 | TGTCGAGACTCTCAGGTTTG |

**Suppl. Table 2:** Primers used for the preparation of NGS samples.

| Primer | Sequence (5ʹ→3ʹ) |
| --- | --- |
| NGS FucT fw1 | CCTCTCTATGGGCAGTCGGTGATATGGCGCAATTGGTTGCCTC |
| NGS FucT fw2 | CCTCTCTATGGGCAGTCGGTGATCCATGCTATGCGCTTGTTAC |
| NGS XylT fw1 | CCTCTCTATGGGCAGTCGGTGATTCTCTTCCCACCCTGATCAC |
| NGS XylT fw2 | CCTCTCTATGGGCAGTCGGTGATGACGGCGGATGGTTTAGGTG |
| NGS XylT1 fw3 | CCTCTCTATGGGCAGTCGGTGATTTACCGGGAGGTGCGATTTC |
| NGS XylT2 fw3 | CCTCTCTATGGGCAGTCGGTGATATGATGAGGTGCCCGCGTTC |
| NGS FucT rev1 a | CCATCTCATCCCTGCGTGTCTCCGACTCAGCTCCACCTGTCCAGGCCCATGAAC |
| NGS FucT rev1 b | CCATCTCATCCCTGCGTGTCTCCGACTCAGTGCAACCTGTCCAGGCCCATGAAC |
| NGS FucT rev1 c | CCATCTCATCCCTGCGTGTCTCCGACTCAGACTAACCTGTCCAGGCCCATGAAC |
| NGS FucT rev2 d | CCATCTCATCCCTGCGTGTCTCCGACTCAGCAGATACCCACCGTCGTGCCAAAG |
| NGS FucT rev2 e | CCATCTCATCCCTGCGTGTCTCCGACTCAGAACTTACCCACCGTCGTGCCAAAG |
| NGS FucT rev2 f | CCATCTCATCCCTGCGTGTCTCCGACTCAGGCGTTACCCACCGTCGTGCCAAAG |
| NGS XylT1 rev1 g | CCATCTCATCCCTGCGTGTCTCCGACTCAGCGATTAAACCATCCGCCGTCACCG |
| NGS XylT1 rev1 h | CCATCTCATCCCTGCGTGTCTCCGACTCAGGTAATAAACCATCCGCCGTCACCG |
| NGS XylT1 rev1 i | CCATCTCATCCCTGCGTGTCTCCGACTCAGAGGCTAAACCATCCGCCGTCACCG |
| NGS XylT2 rev1 j | CCATCTCATCCCTGCGTGTCTCCGACTCAGGATCTAAACCATCCGCCGTCTCCG |
| NGS XylT2 rev1 k | CCATCTCATCCCTGCGTGTCTCCGACTCAGTCACTAAACCATCCGCCGTCTCCG |
| NGS XylT2 rev1 l | CCATCTCATCCCTGCGTGTCTCCGACTCAGTGCGATAAACCATCCGCCGTCTCCG |
| NGS XylT rev2 m | CCATCTCATCCCTGCGTGTCTCCGACTCAGCGCTTCTCACGCATAGTGTGCCTTG |
| NGS XylT rev2 n | CCATCTCATCCCTGCGTGTCTCCGACTCAGTCACCCTCACGCATAGTGTGCCTTG |
| NGS XylT1 rev3 o | CCATCTCATCCCTGCGTGTCTCCGACTCAGCTAGCGGCATCTAGTCGGGATATTC |
| NGS XylT2 rev3 p | CCATCTCATCCCTGCGTGTCTCCGACTCAGACAAACTGCCAACATTCACAAAGTTC |

**Suppl. Table 3**: Mutation frequencies of gRNAs tested by transient expression as determined by NGS.

| gRNA | Sequence (5ʹ→3ʹ) | Mutation frequency (%) |
| --- | --- | --- |
| F3 | GTTCCAAACTTACATCCTA**TGG** | <0.1 |
| F4 | GTAGCCGAAATGCTGCATC**AGG** | 2.1 |
| F5 | GGCACAGCTAGTGTGCTA**CGG** | <0.1 |
| X1 | GAGACCAAGGGAGGTAGGA**GGG** | 0.8 |
| X2 | GCGAGGGTTACTTCGGTAA**TGG** | 0.1 |
| X6 | GGAGGGCAAAATAGGCCA**AGG** | 6.0 |

**Suppl. Table 4:** Mutations in selected X-KO and F-KO T_0_ lines as identified by Sanger sequencing of PCR amplicons. The lengths of the mutations are indicated, as well as the type (deletion –, insertion +). Biallelic and homozygous mutations are shown in the format mut/mut. Here, numbers in parentheses (mut,mut) indicate a combination of deletions and insertions at the target site. Mosaic indicates mutation patterns that cannot be allocated to two germinally mutated alleles and have arisen in the somatic tissue.

| **Line** | **XylT 1** | **XylT 2** | **FucT 1** | **FucT 2** | **FucT 3** | **FucT 4** |
| --- | --- | --- | --- | --- | --- | --- |
| X-KO #1 | mosaic | mosaic | n/a | n/a | n/a | n/a |
| X-KO #8 | mosaic | mosaic | n/a | n/a | n/a | n/a |
| X-KO #10 | -1 | +1 | n/a | n/a | n/a | n/a |
| X-KO #12 | mosaic | +1/+1 | n/a | n/a | n/a | n/a |
| X-KO #17 | mosaic | mosaic | n/a | n/a | n/a | n/a |
| X-KO #29 | mosaic | +4/+5 | n/a | n/a | n/a | n/a |
| X-KO #34 | +1/-36 | mosaic | n/a | n/a | n/a | n/a |
| X-KO #40 | mosaic | Biall. long deletion | n/a | n/a | n/a | n/a |
| X-KO #43 | +1 | +1/+1 | n/a | n/a | n/a | n/a |
| X-KO #44 | -2 | +1/-1 | n/a | n/a | n/a | n/a |
| F-KO #6 | n/a | n/a | +1/-1 | mosaic | Mosaic | mosaic |
| F-KO #10 | n/a | n/a | +1/(-1,+8) | -2/-8 | mosaic | mosaic |
| F-KO #22 | n/a | n/a | mosaic | +1/(-1,+15) | +1 | +1 |
| F-KO #27 | n/a | n/a | (+1,-4)/(+1,-5) | +1/-5 | -1/-1 | +1/-1 |
| F-KO #35 | n/a | n/a | -371/-371 | -1 | +1 | +1/+1 |
| F-KO #37 | n/a | n/a | mosaic | mosaic | mosaic | mosaic |

**Suppl. Table 5:** Primers for high resolution melt analysis of T_1_ plants.

| Name | Sequence (5ʹ→3ʹ) |
| --- | --- |
| FucT1-4 HRMA fw | TCTCAATTTGATGGCAGTTG |
| FucT1/2-4 HRMA rev | ACCAGCTACCAGAGACTGAAAG |
| FucT2-4 HRMA fw | TCTCAATTTGATGGCAGTAG |
| FucT3/4-4 HRMA fw | TCATGGCAGTGGACAAAGTG |
| FucT3-4 HRMA rev | CATCGGAAAGTAAGTGTAAAGAG |
| FucT4-4 HRMA rev | TGCAGAACCAAAATTAACAG |
| XylT1 HRMA fw | CCACTCTTCAATCACTTCTC |
| XylT1 HRMA rev | CCGAATTTCCGGTGAAACTC |
| XylT2 HRMA fw | CTTCAATCACTTCCCAATATTCC |
| XylT2 HRMA rev | CGAATTTCCGGTGAAGCTC |

**Suppl. Table 6:** Mutations in F-KO T_1_ plants selected by dot blot**.** The lengths of the mutations are indicated, as well as the type (deletion –, insertion +). Biallelic mutations are shown in the format mut/mut. Numbers in parentheses (mut,mut) indicate multiple mutations in one allele. Abbreviations: wt = wild type, het = heterozygous.

| **Line** | ***FucT* 1** | ***FucT* 2** | ***FucT* 3** | ***Fuct* 4** |
| --- | --- | --- | --- | --- |
| F-KO #35-1 | -1/-1 | -1 | +1/+1 | +1/+1 |
| F-KO #35-2 | -371/-371 | wt | +1/+1 | +1/+1 |
| F-KO #35-3 | -1/-2 | -1 | +1 | +1/+1 |
| F-KO #35-4 | -371/-371 | wt | +1 | +1/+1 |
| F-KO #35-5 | -371/-371 | wt | +1/+1 | +1/+1 |
| F-KO #35-6 | -371/-371 | wt | +1/+1 | +1/+1 |
| F-KO #35-7 | -31/-2 | -1 | wt | +1/+1 |
| F-KO #35-8 | -371/-371 | -1 | +1/+1 | +1/+1 |
| F-KO #35-9 | -371/-371 | -1 | +1 | +1/+1 |
| F-KO #35-10 | -371/-1 | wt | +1 | +1/+1 |
| F-KO #35-11 | -371/-371 | -1 | +1/+1 | +1/+1 |
| F-KO #35-12 | -371/-371 | wt | +1 | +1/+1 |
| F-KO #35-13 | -371/-371 | -1 | +1 | +1/+1 |
| F-KO #35-14 | -371/-371 | -1 | +1/+1 | +1/+1 |
| F-KO #35-15 | -31/-1 | -1 | +1/+1 | +1/+1 |
| F-KO #6-3 | +1/+1 | +1 | +1 | +1/-6 |
| F-KO #6-5 | +1/+1 | -1 | (+1,-8) het | mosaic |
| F-KO #6-10 | -1/-6 | -1 | +1 | +1/+1 |
| F-KO #6-15 | +1/-6 | +1 | -3 | -1/-1 |


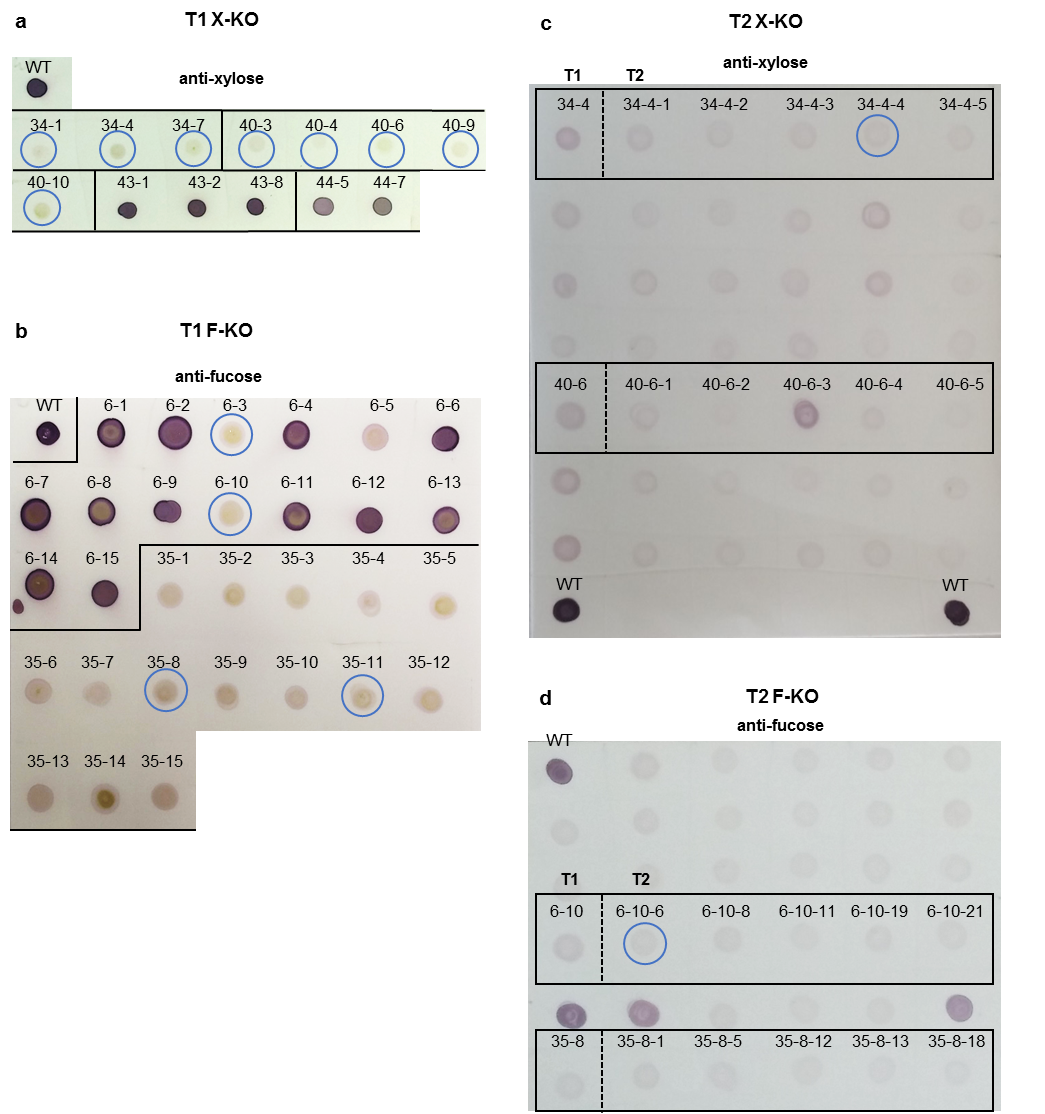


**Suppl. Figure 1: Dot blots of crude leaf extracts from T_1_ and T_2_ X-KO and F-KO plants** Plants selected for further analysis are marked by a blue circle. Primary antibodies (a, c) rabbit-anti- β1,2-xylose (diluted 1:5000, ~2 µg/membrane) and (b, d) rabbit-anti-α1,3-fucose (diluted 1:10,000, ~1 µg/membrane) were detected with a secondary goat-anti-rabbit H+L AP-labeled antibody. Wild-type leaf extract was used as a positive control for both primary antibodies. (a) Eight of 13 tested T_1_ X-KO plant samples were not detected by the xylose-specific antibody. (b) Seventeen of 30 tested T_1_ F-KO plant samples showed weak or absent signals. (c) T_2_ progeny of two selected X-KO lines showed weak or absent reactions with the primary antibody. (d) T_2_ progeny of two selected F-KO lines were tested with the fucose-specific antibody using wild-type and T_1_ sample as controls. Note that plants 35-8 and 35-8-13 are mutated in 7 and 8 alleles of the four *FucT* genes, respectively, and show a similar signal intensity. Only selected lines are labelled. Descendants of a single line are limited by a solid black line. Dotted lines separate the T2 samples from the corresponding T1 samples, loaded for comparison.


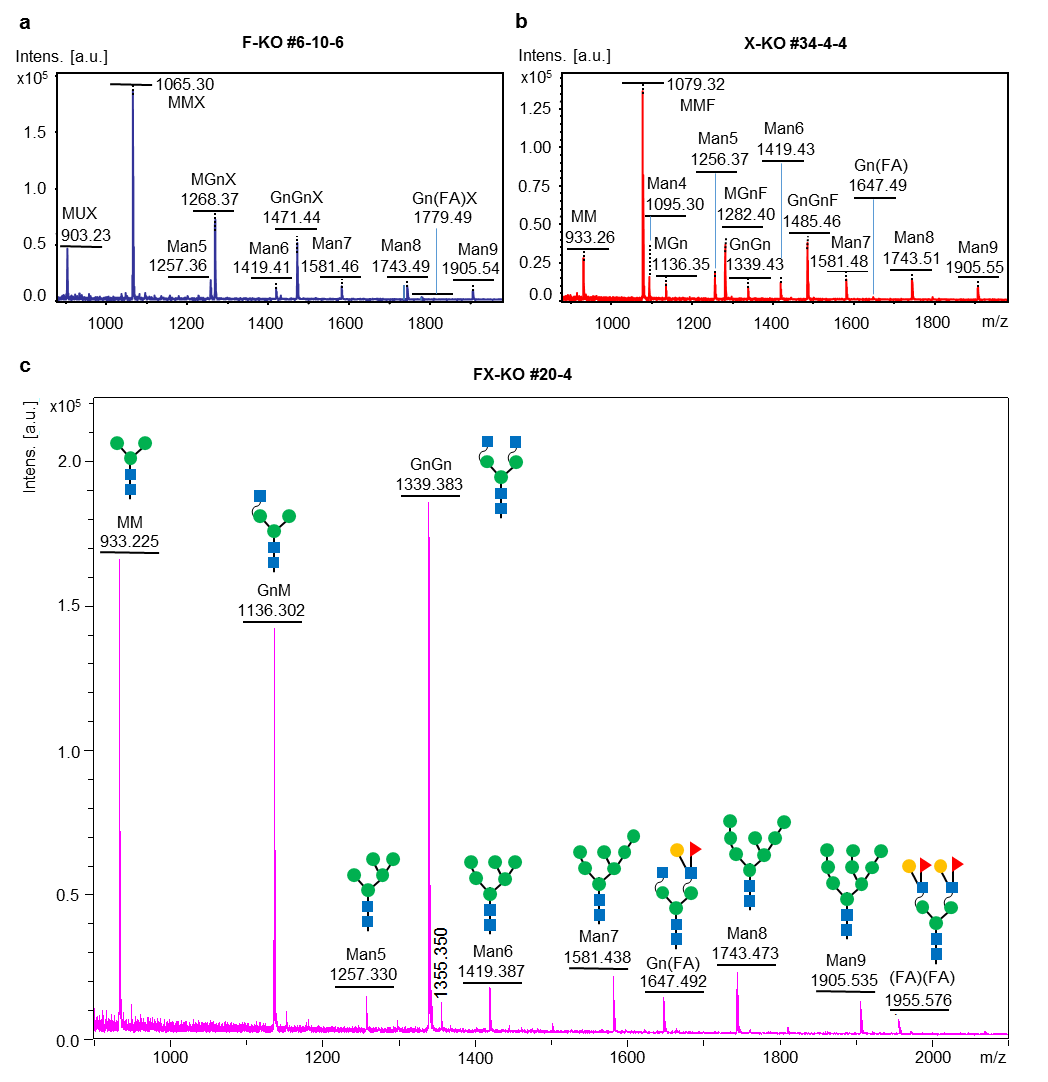


**Suppl. Figure 2: Leaf proteome N-glycan analysis for the T2 lines F-KO #6-10-6 and X-KO #34-4-4 and the F2 line FX-KO #20-4.** (**a**) MALDI-TOF MS analysis of N-glycans released from the leaf proteome of F-KO #6-10-6. No α-1,3-fucosylated N-glycans were found. (**b**) MALDI-TOF MS analysis of N-glycans released from the leaf proteome of X-KO #34-4-4. No β-1,2-xylosylated N-glycans were found. (**c**) MALDI-TOF analysis of N-glycans released from the leaf proteome of FX-KO #20-4. No α-1,3-fucosylated and β-1,2-xylosylated N-glycans were found. Glycan acronyms are based on proglycan nomenclature (<http://www.proglycan.com>). A MALDI-TOF spectrogram of a N. benthamiana wild type sample prepared using the same protocol can be found in Strasser et al. (2008), Figure 2a.


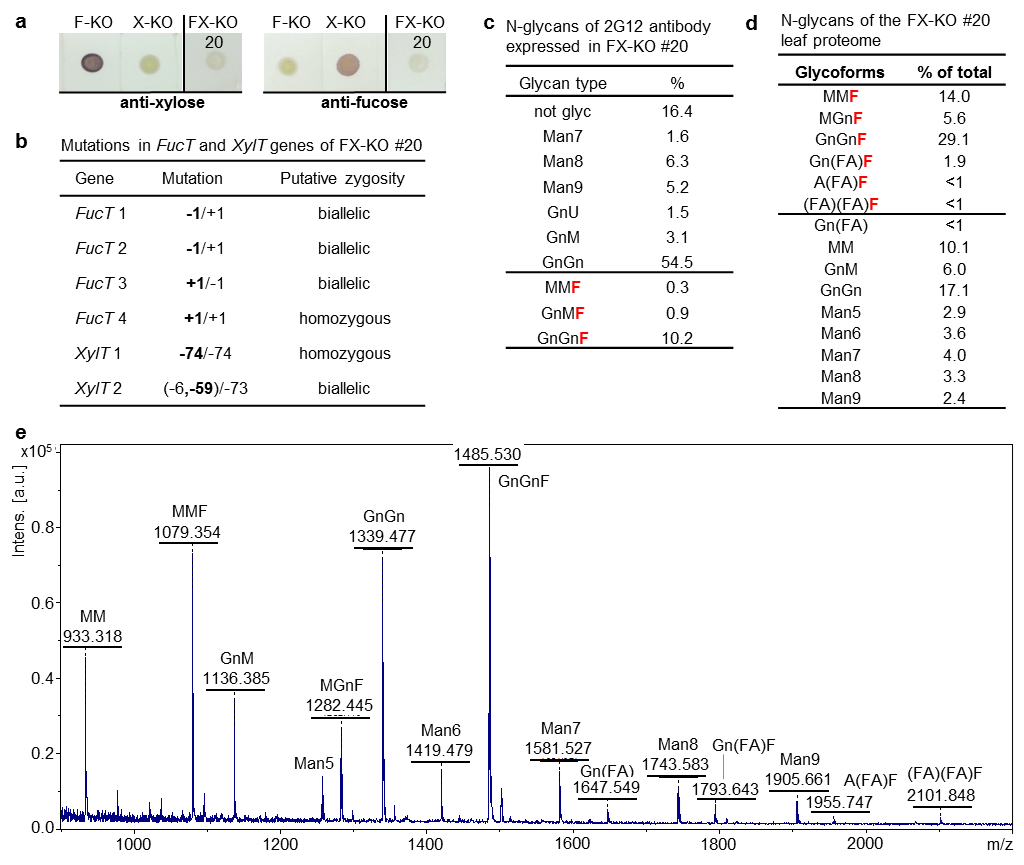


**Suppl. Figure 3: Analysis of the hybrid F1 generation line FX-KO #20 obtained by crossing F-KO #6-10-6 and X-KO #34-4-4.** (a) Dot blots with xylose-specific antibody (left) and core-fucose-specific antibody (right), using confirmed F-KO and X-KO samples as positive and negative controls. FX-KO #20 tested negative in both blots. (b) Sanger sequencing results of FX-KO #20 revealing one inherited allele in bold, and one newly (somatic or germinal) mutated allele per gene. (c) LC-ESI-MS glycopeptide analysis of the antibody 2G12 transiently expressed in FX-KO #20. No β‑1,2-xylosylated N-glycans were found, but >11% was represented by α-1,3-fucosylated N-glycans. (d + e) MALDI-TOF results for total endogenous leaf protein N-glycan analysis. No β-1,2- xylosylated N-glycans were found, but ~50% of the total was represented by α-1,3-fucosylated N-glycans.


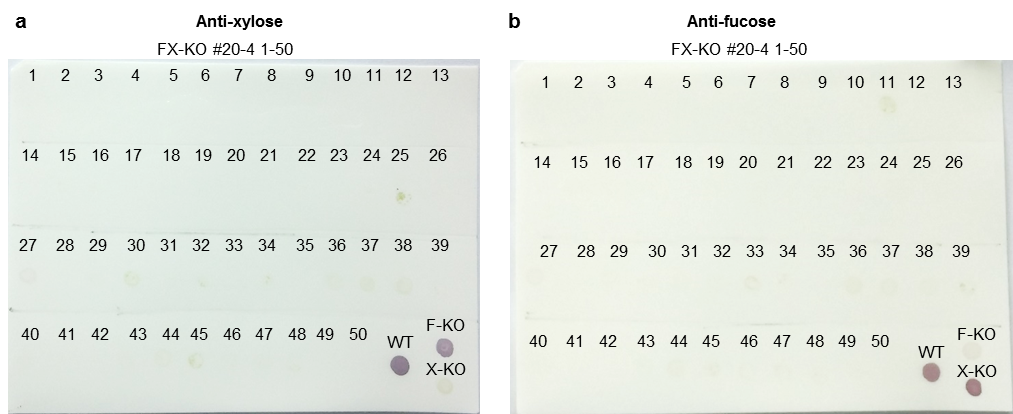


**Suppl. Figure 4: Dot blots of 50 F3 progeny plants of FX-KO #20-4.** Extract from N. benthamiana wild type, F-KO #6-10-6 and X-KO #34-4-4 were used as controls. (**a**) Anti-xylose blot. None of the 50 F3 plants tested positive. (**b**) Anti-fucose blot. None of the 50 F3 plants tested positive.


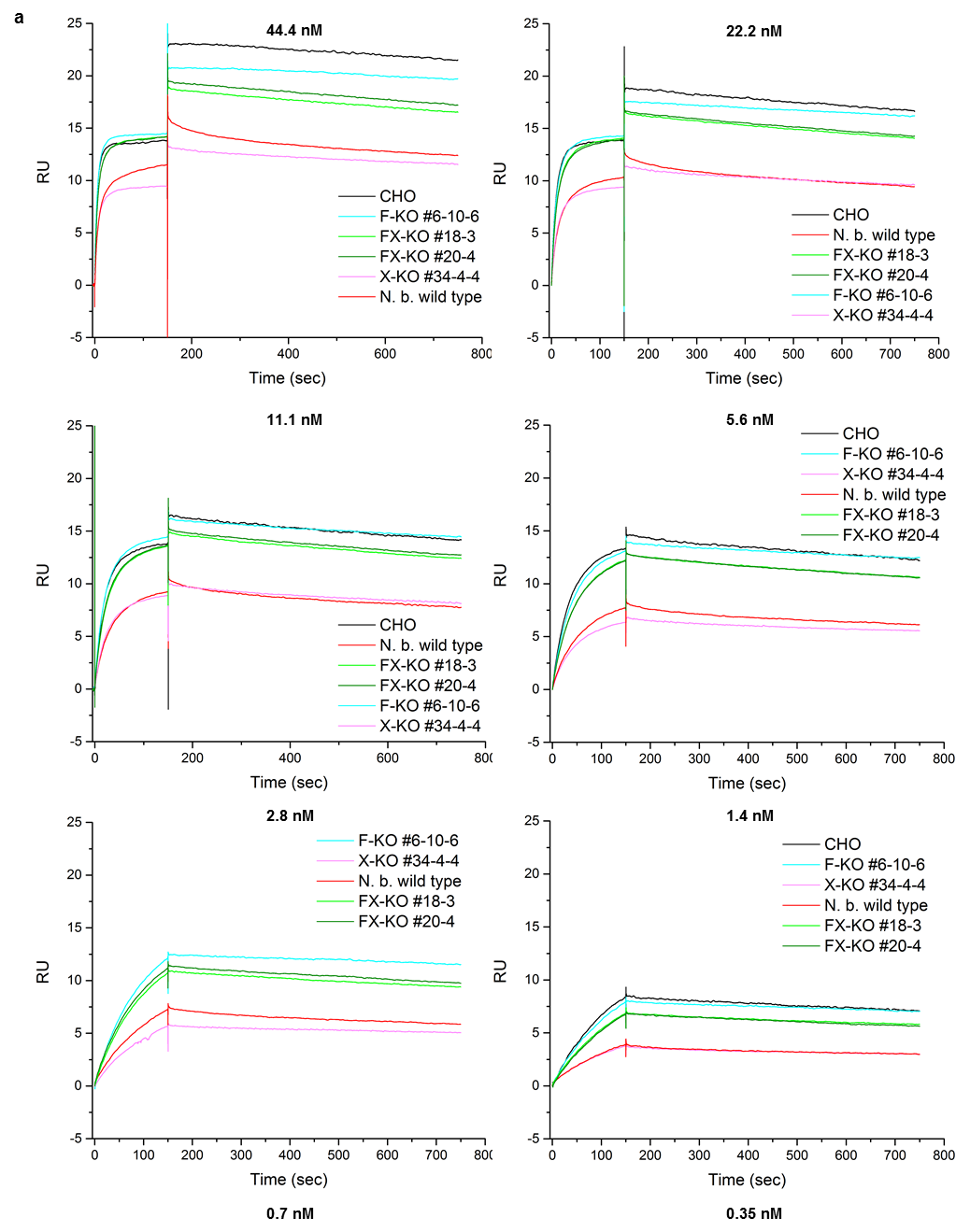


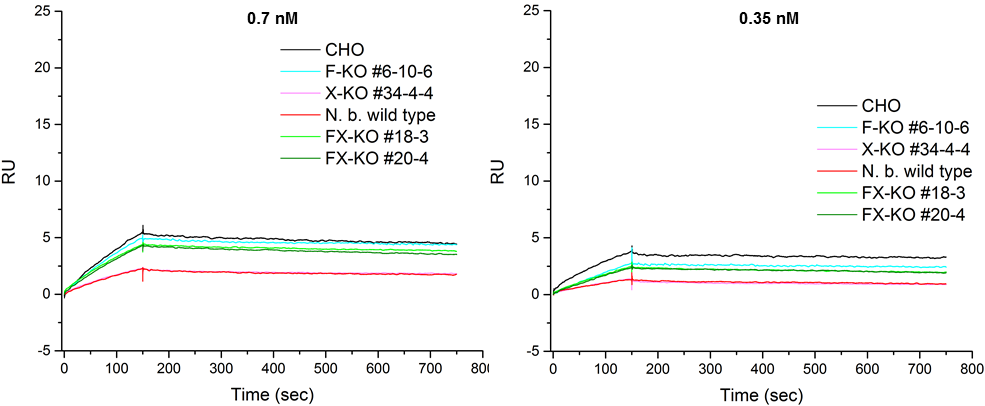


***
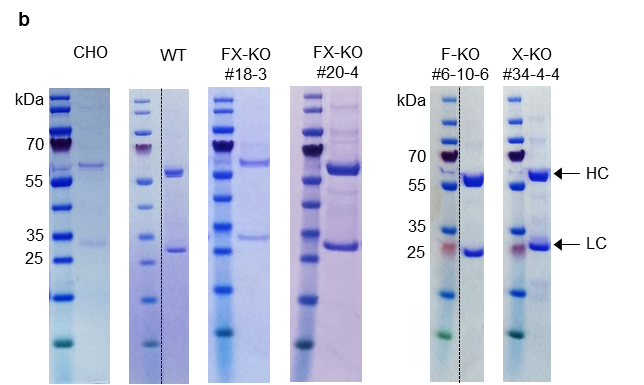
***

***Suppl. Figure 5: Surface plasmon resonance assay for CD64 binding using antibody 2G12 glycovariants expressed in wild-type N. benthamiana, X-KO #34-4-4, F-KO #6-10-6, FX-KO #18-3 and FX-KO #20-4 lines, and CHO cells (mammalian N-glycosylation).***  *Equal amounts of each antibody (50 RU) were bound to a protein A surface, and eight concentrations of human CD64 receptor (44.4–0.35 nM) were used in a multicycle kinetic to compare the CD64-binding behavior of the different glycovariants. The results shown here were obtained from the original spectrograms, corrected with the actual RU value (corrected RU_kinetic_=raw RU_kinetic_*(50 RU_antibody_/actual RU_antibody_), and normalized to start at the axis origin (x=0/y=0). (****a****) Obtained 2G12-CD64 binding curves categorized by CD64 concentration. For 2.8 nM, the data point for CHO 2G12 was excluded because the curve did not fit the overall picture and indicated a technical problem, most likely an air bubble that distorted the signal. For all concentrations, wild-type 2G12 and X‑KO 2G12 show very similar binding behavior, as do CHO and F-KO 2G12. The two FX-KO samples produced in two different lines exhibit the same binding behavior towards CD64, as expected because of their comparable N-glycan profiles. (****b****) Coomassie-stained SDS-PAGE gels of the CHO control and the plant-produced, purified antibodies. While the gels show different amounts of antibody, the SPR assays were conducted using the same amount for each sample.*

**Suppl. Appendix S1: Next generation sequencing**

To generate amplicons for IonTorrent sequencing, a one-step PCR strategy was applied using gene-specific primer pairs with the required adapters and barcodes already attached (**Suppl. Table 2**). The target regions were amplified from genomic DNA extracted from infiltrated leaf material, successful amplification was verified by gel electrophoresis, and the desired products were purified from the gel using the NucleoSpin Gel and PCR Clean-up kit. The products were quantified by capillary electrophoresis using the Agilent DNA 1000 Kit and the Agilent Bioanalyzer 2100 (Agilent Technologies, Santa Clara, USA). All samples were diluted to 100 pM, and 2 µL of each was added to a sample pool that was processed with the Ion Library Equalizer kit (Thermo Fisher Scientific) to further purify the amplicons and dilute them to a final concentration of 26 pM. The amplicon library was then processed with the Ion PGM Sequencing 400 kit, and sequenced on an Ion 318 chip in an Ion Personal Genome Machine (Thermo Fisher Scientific). The resulting barcoded sequences were aligned against the corresponding template sequence using the DNASTAR Lasergene software package (DNASTAR, Madison, USA), and analyzed for mutations at the gRNA target sites.

**Suppl. Appendix S2: Analysis of chromatograms obtained by simultaneous Sanger sequencing of both alleles of a gene**

To determine the mutation status of a target gene, gene-specific primers were used to amplify the region of interest from genomic DNA. The genome of *N. benthamiana* is allotetraploid (amphidiploid), which means that it is the hybridization product of the complete diploid chromosome sets of two parents. Every gene can be allocated to one or the other parental genome, and has two copies, but will also have a homologue in the other chromosome set. In terms of the genes relevant here, this means that we have the diploid *XylT* 1 gene from one parental genome, and the diploid *XylT* 2 gene from the other parental gene. Their sequences are similar enough to be targeted by the same gRNAs, but different enough to allow the design of gene-specific primer pairs that will amplify one gene variant but not the other. The same principle applies to *FucT* 1 and *FucT* 2, as well as *FucT* 3 and *FucT* 4. We exploited the diploid nature of the individual genes and the gene-specific primer pairs to determine the mutation status of each targeted gene.

Both alleles of a given gene were amplified from genomic DNA, the PCR amplicons purified and sequenced using the Sanger method. Depending on the mutation status of the two alleles, there are five possible outcomes:

- Wild type: neither allele is mutated (one trace)
- Heterozygous: one allele is wild type, the other is mutated (two overlapping traces)
- Biallelic: both alleles are mutated but the mutations are distinct (two overlapping traces)
- Homozygous: both alleles are mutated in the same manner (one trace)
- Mosaic: multiple mutations are found in the sample (multiple overlapping traces)

Combinations are also possible, e.g. if one allele was mutated early in plant development and all cells in the plant carry this mutation, and the other allele shows mosaic/somatic mutations induced later in development that will probably not be transmitted to the next generation.

The Sanger sequencing results were aligned with the corresponding template in CloneManager, and the chromatograms scanned by eye for overlapping traces downstream of the Cas9 cut sites. Suppl. Fig. 5 shows examples of chromatograms for a wild-type, heterozygous and homozygous samples.


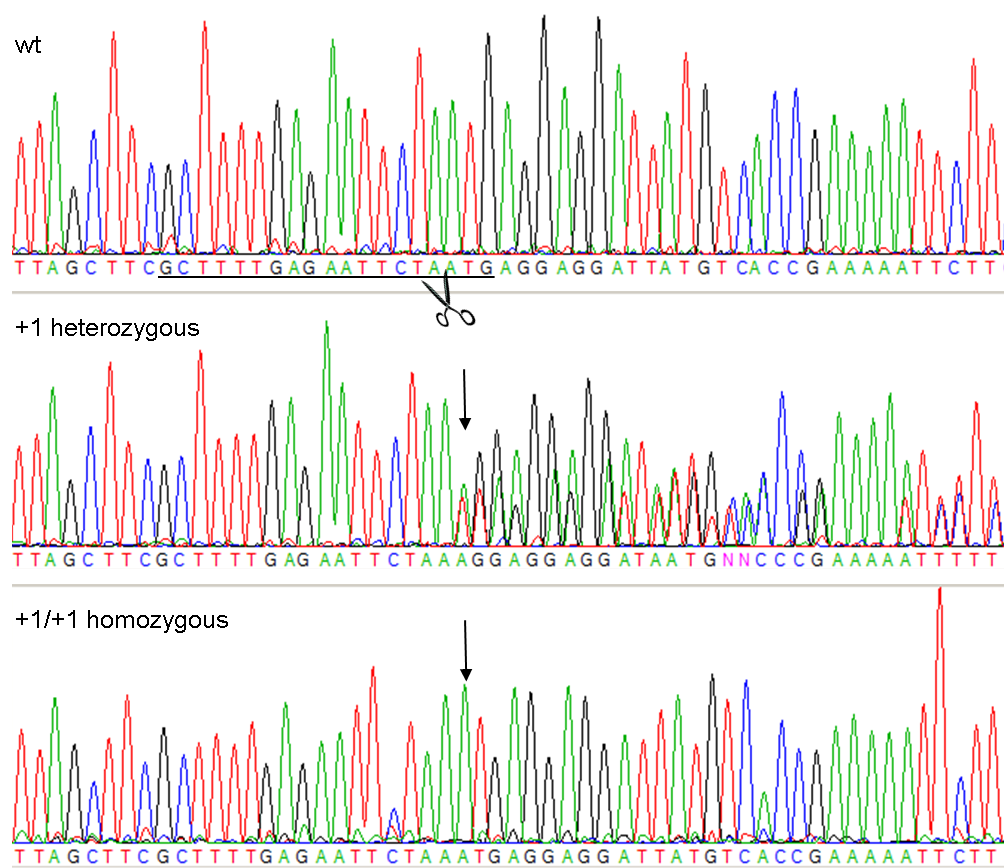


***Suppl. Figure 6: Chromatograms obtained by Sanger sequencing of FucT 3 exon 4 in a wild-type plant, a heterozygous mutant, and a homozygous mutant.*** *In the wild-type chromatogram, the spacer sequence upstream of the AGG PAM is underlined in black, and the Cas9 cut site is indicated using a scissor symbol. In the heterozygous sample, one allele has a +1 insertion (A) and from the cut site onwards, two overlapping chromatogram traces can be observed. In the homozygous sample, both alleles have a +1 insertion (A), resulting in a single chromatogram trace, because the sequences are identical.*

Short or homozygous mutations can be identified based on the chromatograms, but longer and more complex mutations (e.g. biallelic mutations) require further analysis. Usually the presence or absence of wild-type sequences in a chromatogram can easily be determined, but supporting the proposed genotype with a bioinformatics tool gives clearer results. For every mutated sample, we routinely used TIDE (Brinkman et al., 2014), an online tool that Tracks Indels by DEcomposition based on a control and a sample chromatogram and the gRNA sequence supplied by the user. Insertions and deletions of up to 50 nucleotides can be identified by the software. For samples with longer, biallelic deletions that could not be identified with TIDE or in CloneManager, the PCR product in question was cloned into TOPO vectors and a number of clones were sequenced independently. However, for the majority of samples processed in this study, direct sequencing of the PCR product was sufficient and proved a simple and straightforward approach for the genotyping of large numbers of plants.

**Suppl. Appendix S3: N-glycan analysis of antibody heavy chain glycopeptides by LC-ESI-MS**

The heavy chain samples were S-alkylated with iodoacetamide and digested with trypsin (Promega) before loading onto a BioBasic C18 column (BioBasic-18, 150 x 0.32 mm, 5 µm; Thermo Fisher Scientific) in 65 mM ammonium formiate (buffer A) with 100% acetonitrile as buffer B. A gradient from 5% to 32% B in 35 min was applied, followed by a gradient from 32% B to 75% B in 15 min to elute large peptides, at a flow rate of 6 µL/min. Peptides were detected using a QTOF-MS (Bruker maXis 4G) equipped with the standard ESI source in positive ion DDA mode, which means switching to MS/MS mode for eluting peaks. Mass spectra were recorded in the range 150–2200 Da, and the three highest peaks were selected for fragmentation. The instrument was calibrated using ESI calibration mixture (Agilent Biotechnologies). Manual glycopeptide searches were carried out using DataAnalysis v4.0 (Bruker). For the quantification of the different glycoforms, the peak areas of extracted ion chromatograms representing the first four isotopic peaks were summed, using the quantification software Quant Analysis (Bruker). This work was carried out by Clemens Grünwald-Gruber and Friedrich Altmann (BOKU, Vienna, Austria).

**Suppl. Appendix S4: Dot blots of knock-out candidate lines**

Selected plant lines were tested for the presence of α-1,3-fucose or β-1,2-xylose in N-glycans by dot blot using polyclonal affinity-purified rabbit sera specific for each residue (Agrisera AB, Umea, Sweden). A protein extract was made from ~100 mg frozen leaf material by extracting it in two volumes of phosphate buffered saline (137 mM NaCl, 2.7 mM KCl, 8.1 mM Na_2_HPO_4_, 1.5 mM KH_2_PO_4_, pH 7.4) containing 500 mM NaCl and 10 mM Na_2_S_2_O_5_, and centrifuging for 10 min at 16,100 x *g* and 4°C. The supernatant was transferred to a fresh tube, and 2 µL was used for blotting. The nitrocellulose membrane (Thermo Fisher Scientific) was placed on a filter paper soaked with TBS-T (20 mM Tris, 150 mM NaCl, 0.05% (v/v) Tween-20, pH 7.5) on top of a dry filter paper. The protein extracts were spotted onto the membrane with a micropipette and left to dry for 90 min at room temperature. After blocking with 2% skimmed milk powder dissolved in TBS-T for 30 min at room temperature under agitation, the membrane was incubated with the primary antibody for 30 min at room temperature (anti-α-1,3-fucose 1:10,000, anti-β-1,2-xylose 1:5000 in TBS-T). The membrane was then washed three times for 10 min each in TBS-T before the secondary goat-anti-rabbit H+L alkaline phosphatase (AP)-conjugated antibody (Jackson ImmunoResearch Laboratories, Westgrove, USA) was added, diluted 1:5000 in TBS-T, and incubated for 1 h. After three 10-min washes as above, the AP substrate (100 µL BCIP/NBT) was added in 10 mL AP buffer (100 mM Tris-HCl, 100 mM NaCl, 5 mM MgCl_2_, pH 9.6) and the blot was developed in the dark at room temperature.

Strasser, R., Stadlmann, J., Schähs, M., Stiegler, G., Quendler, H., Mach, L., Glössl, J., Weterings, K., Pabst, M. and Steinkellner, H. (2008) Generation of glyco-engineered *Nicotiana benthamiana* for the production of monoclonal antibodies with a homogeneous human-like N-glycan structure. *Plant Biotechnology Journal* **6**, 392-402.
